# Photocontrolling the Enantioselectivity of a Phosphotriesterase via Incorporation of a Light-Responsive Unnatural Amino Acid

**DOI:** 10.1021/jacsau.4c01106

**Published:** 2025-02-05

**Authors:** Caroline Hiefinger, Gabriel Zinner, Torben F. Fürtges, Tamari Narindoshvili, Sebastian Schindler, Astrid Bruckmann, Till Rudack, Frank M. Raushel, Reinhard Sterner

**Affiliations:** † Institute of Biophysics and Physical Biochemistry, Regensburg Center for Biochemistry, University of Regensburg, D-93053 Regensburg, Germany; ‡ Department of Chemistry, Texas A&M University, College Station, Texas 77843-3255, United States; § Institute of Biochemistry, Genetics and Microbiology, Regensburg Center for Biochemistry, University of Regensburg, D-93053 Regensburg, Germany

**Keywords:** genetic code expansion, molecular dynamics simulations, organophosphate hydrolysis, phosphotriesterase, photocage, stereoselectivity switch, unnatural amino acid

## Abstract

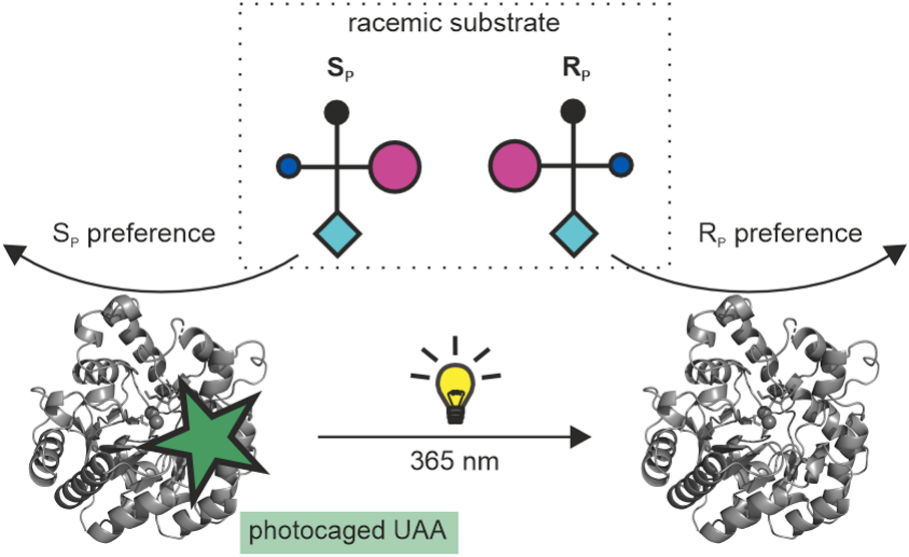

The external control of catalytic activity and substrate specificity of enzymes by light has aroused great interest in the fields of biocatalysis and pharmacology. Going beyond, we have attempted to photocontrol enzyme stereoselectivity on the example of phosphotriesterase (PTE), which is capable of hydrolyzing a wide variety of racemic organophosphorus substrates where one of two enantiomers is often highly toxic. To pursue this goal, the photocaged unnatural amino acid *o*-nitrobenzyl-l-tyrosine (ONBY) was incorporated by genetic code expansion at the large subsite of the active center, together with additional mutations at the small subsite. The stereoselectivities of the resulting PTE variants were tested with the achiral control substrate paraoxon and four different racemic substrates, which contained a *p*-nitrophenol leaving group in combination with either methyl-phenyl, ethyl-phenyl, methyl-cyclohexyl, or ethyl-cyclohexyl substituents. Comparison of the enantioselectivities (*k*_cat_/*K*_M_ for S_p_ divided by *k*_cat_/*K*_M_ for R_p_) before and after decaging of ONBY using irradiation revealed the desired photoinduced inversion of enantioselectivity for three of the variants: PTE_I106A-H257ONBY exhibited a 43-fold stereoselectivity switch for the methyl-phenyl substrate, PTE_I106A-F132A-H257ONBY a 184-fold stereoselectivity switch for the methyl-cyclohexyl substrate, and PTE_I106A-F132A-S308A-H257ONBY a 52-fold and a 57-fold stereoselectivity switch for the methyl-cyclohexyl and the ethyl-cyclohexyl substrates. A computational analysis including molecular dynamics simulations and docking showed that a complicated interplay between steric constraints and specific enzyme–substrate interactions is responsible for the observed effects. Our findings significantly broaden the scope of possibilities for the spatiotemporal control of enantioselective transformations using light in biocatalytic systems.

## Introduction

The manipulation and control of enzymatic activity is an important goal that has been pursued in a multitude of research areas over the past decades. Within this context, stimuli-responsive proteins are an exciting class of biomaterials that have gained increasing attention due to their tremendous potential for various applications. A specific stimulus can be employed to induce alterations in the biochemical or biophysical properties, such as oligomerization state, three-dimensional structure, conformational dynamics, or ligand binding, thereby allowing for the effective on-demand control of enzymes. To date, a variety of physical and chemical stimuli have been used for this purpose, including temperature, pH, metal ions, magnetic field, ligands, chemical inducers, as well as light.^1−12^ Light has emerged as a powerful stimulus as it offers the advantage of being noninvasive and moreover, its wavelength and intensity can be tuned with high spatiotemporal resolution. Consequently, it represents a versatile tool for the artificial photocontrol of various biological systems and mechanisms.^13−15^

Various strategies have been established over the past decades to create light-responsive enzymes, one of them being genetic code expansion (GCE) where light-sensitive unnatural amino acids (UAAs) are incorporated site-specifically into a protein of interest.^16−18^ Hitherto, many studies have employed such UAAs to realize the regulation of enzymatic activity but also to enable the control of oligomerization, feedback inhibition, substrate binding, and allosteric mechanisms via light.^19−25^

**Scheme 1:**
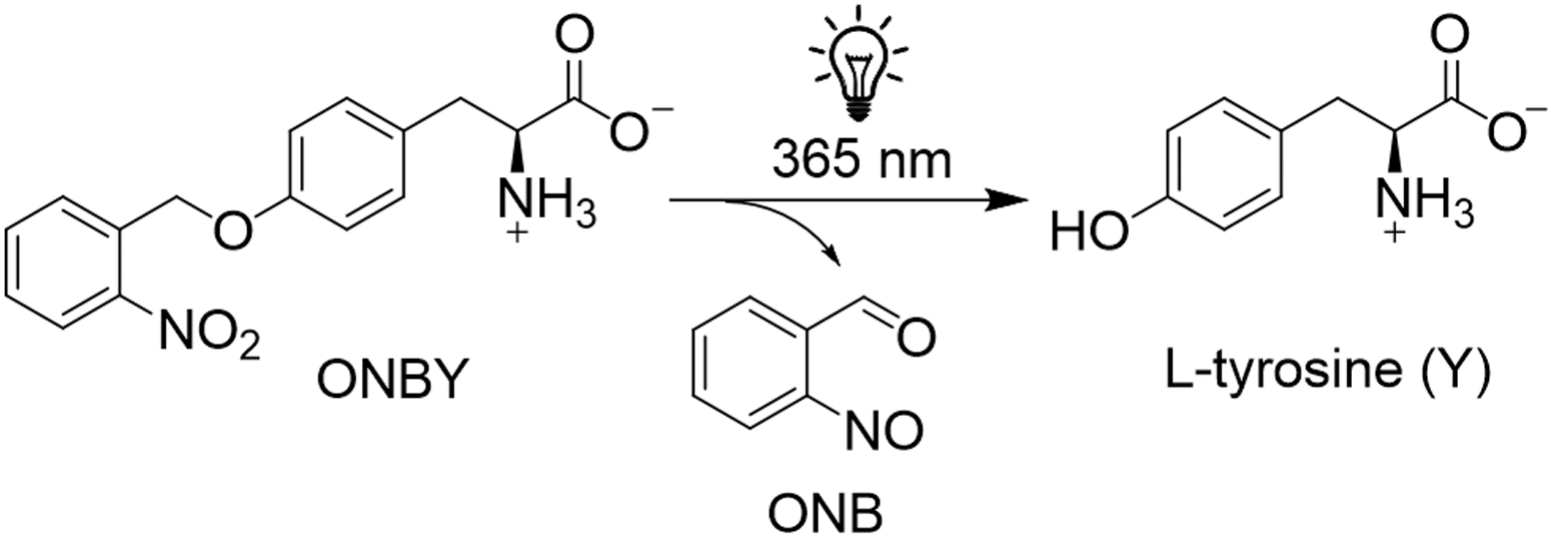
Irreversible Cleavage of the Photocaged UAA ONBY by UV-Light Resulting in *o*-Nitrosobenzyladehyde (ONB) and l-Tyrosine

Among the wide array of biophysical properties that can be targeted to influence enzymes in a desired manner, approaches to control stereoselectivity are of special interest. Particularly, in the fields of pharmacology and biocatalysis, the precision of biochemical processes as well as the avoidance of undesired side reactions is decisive for process outcome and quality.^26^ While most studies have employed point mutations,^27,28^ alterations in stereoselectivity of enzymes such as lipase or chymotrypsin were achieved by means of external stimuli like temperature, reaction medium, solvation thermodynamics, additives, or pressure.^29−34^ However, employing light as a stimulus for high spatiotemporal control of stereoselectivity has only been reported rarely.^35−37^ In most cases, photoresponsive molecules were covalently linked to certain amino acids, which has the drawback of being nonspecific and may vary in different protein batches, resulting in heterogeneous protein populations. Furthermore, chemical modification might partially be incomplete or lead to undesired modification of other proteins during expression.^36^ To circumvent these issues, GCE can be used as a more precise strategy to achieve site-specific modification of an enzyme. The photosensitive UAAs that are commonly incorporated are either photocaged UAAs, that are based on a natural amino acid with an attached caging group^24,38−40^ or photoswitchable UAAs that interconvert between two different isomer forms.^41−44^ Although photoswitchable UAAs offer the advantage of reversibility, photocaged UAAs typically achieve higher light-regulation factors, defined as the ratio of activities before and after irradiation.^21−23^ Furthermore, photoswitches typically do not achieve fully quantitative switching as an equilibrium of isomer distribution is induced upon irradiation, which may revert to the initial state unless irradiation is maintained.^41,43^ In contrast, photocages follow an all-or-nothing principle by cleavage of the caging group upon a single exposure and regenerate a natural amino acid, minimizing interference with the protein’s native structure or dynamics before activation. The most popular and widely used photocaged UAA is *o*-nitrobenzyl-l-tyrosine (ONBY) where *o*-nitrosobenzaldehyde is cleaved after exposure to light of 365 nm resulting in l-tyrosine (Scheme 1).^24^

In the past, ONBY has been extensively used for the photocontrol of enzymes via modulation of catalytic activity, feedback inhibition, oligomerization, phosphorylation, cap methylation, and mRNA translation.^19−21,45−47^ However, photoinduced alterations in substrate stereoselectivity facilitated by incorporation of a light-responsive UAA have not been reported so far.

**Scheme 2:**
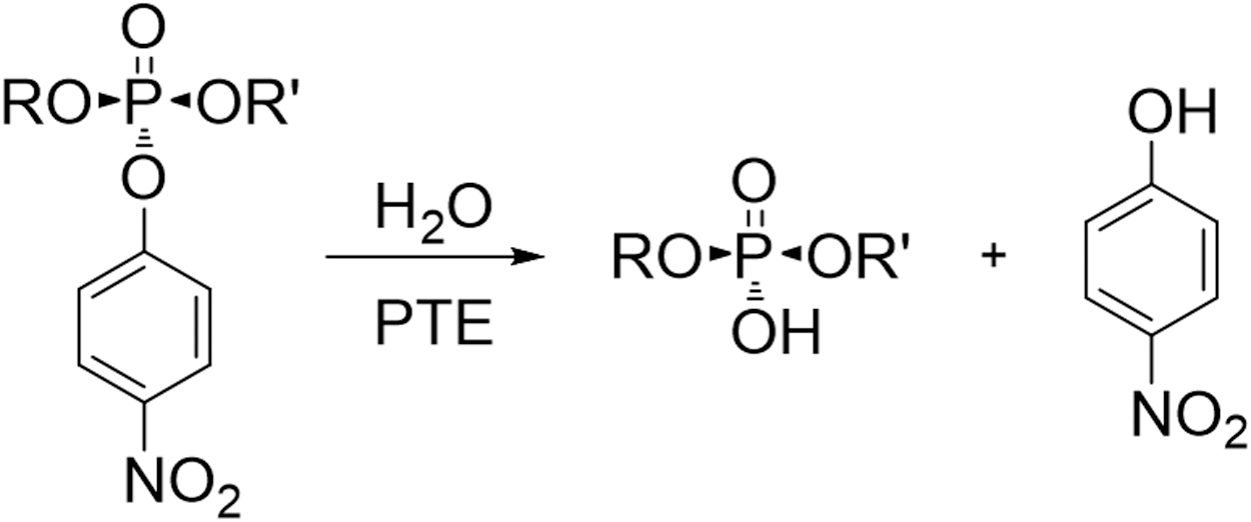
Hydrolysis of *p*-Nitrophenyl-Substituted Phosphotriesters by PTE The reaction yields the respective organophosphate and *p*-nitrophenol.^58^ R and R′ represent alkyl or aryl substituents. If R differs from R′, two stereoisomers (S_P_, R_P_) exist, one of which is the preferred substrate.

In the context of manipulating stereoselectivity, phosphotriesterase (PTE) has emerged a well-suited enzyme system.^48−52^ PTE from *Pseudomonas diminuta* is a homodimeric enzyme belonging to the amidohydrolase superfamily^53,54^ that is capable of hydrolyzing a wide variety of organophosphorus compounds. In the case of chiral substrates, it commonly favors the hydrolysis of one stereoisomer.^55−58^ The best substrate identified to date is paraoxon with a *k*_cat_/*K*_M_ value approaching the diffusion-controlled limit of ∼10^8^ M^–1^ s^–1^.^59^ The overall hydrolysis reaction proceeds via an S_N_2-like mechanism with a net inversion of stereochemistry at the phosphorus center.^60,61^ A generalized reaction scheme for the hydrolysis of a *p*-nitrophenyl substituted phosphotriester is depicted in Scheme 2.

**Figure 1:**
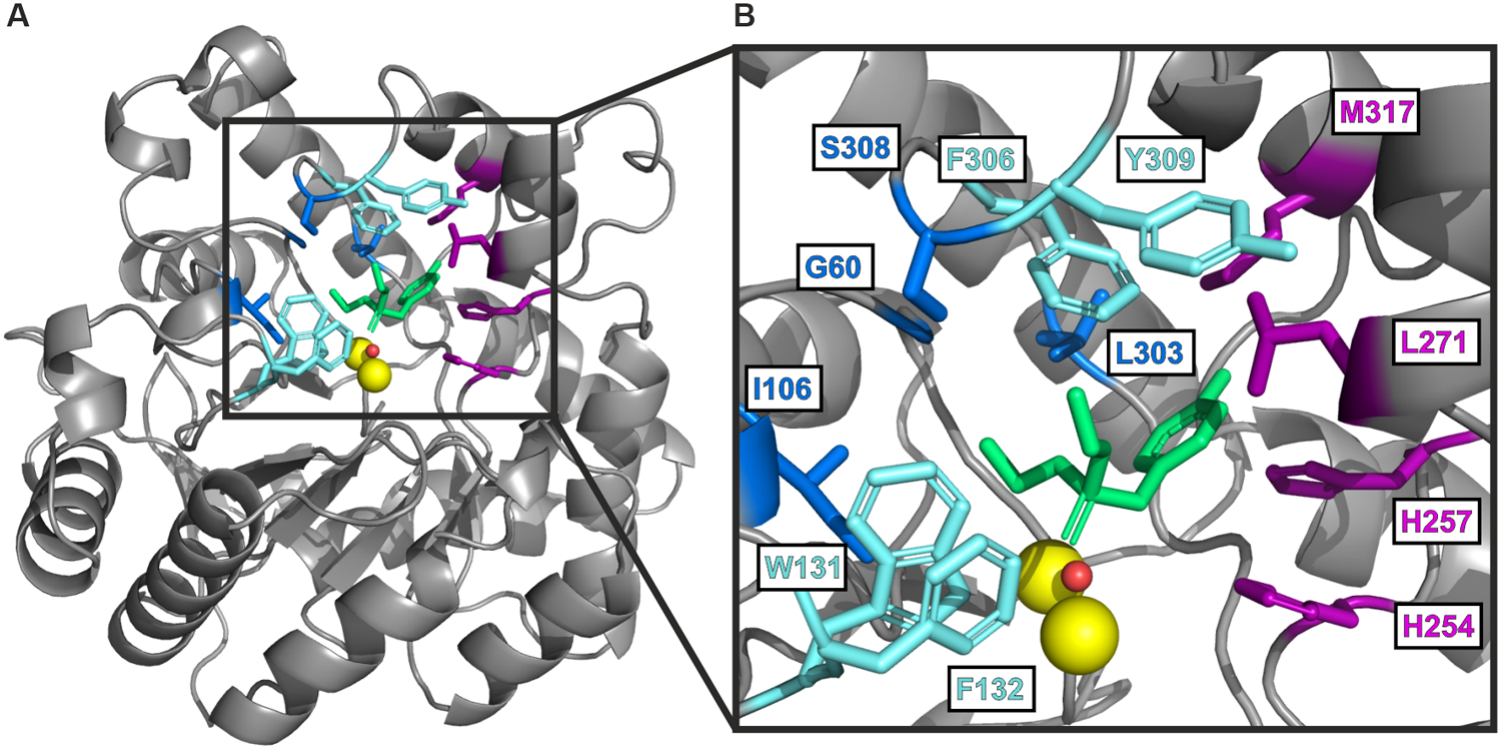
Crystal structure and active site of PTE from *Pseudomonas diminuta.* (A) PTE (PDB: 1dpm)^64^ adopts a (βα)_8_-barrel fold characteristic for enzymes of the amidohydrolase superfamily. For reasons of clarity, only one subunit of the homodimer is shown. (B) Enlarged section of the active site of PTE complexed with two Zn^2+^ ions (yellow), a water molecule (red), and the substrate analog diethyl 4-methylbenzyl phosphonate (green). The small subsite-coordinating residues Gly60, Ile106, Leu303, and Ser308 are highlighted in blue, and the large subsite-coordinating residues His254, His257, Leu271, and Met317 in purple. The leaving group-coordinating residues Trp131, Phe132, Phe306, and Tyr309 in the substrate entrance/exit site are depicted in cyan. Adapted from ref (64). Copyright [1996] American Chemical Society.

The active site of PTE contains a binuclear metal center with two zinc ions that are each coordinated by four histidines (His55, His57, His201, His230) and one aspartate (Asp301).^59,62^ A molecule of solvent and a carboxylated lysine (Lys169) serve as bridging ligands between the two metal ions.^63,64^ The native zinc cations can be substituted with Mn^2+^, Co^2+^, Ni^2+^ or Cd^2+^, or a combination of them without any significant loss of catalytic activity.^65,66^ Importantly, the active site of PTE features three subsites that are decisive for the orientation of the different alkyl and aryl substituents of the substrate (Figure 1).^51,58,64,67,68^ The small subsite is mainly formed by the side chains of Gly60, Ile106, Leu303, and Ser308 while the large subsite is predominantly defined by His254, His257, Leu271 and Met317. The leaving group subsite, which simultaneously is the entrance and exit site of the substrate, encompasses Trp131, Phe132, Phe306, and Tyr309. The different sizes of the small and the large subsites are responsible for the orientation of the substrate within the active site. Consequently, PTE displays a preference for one enantiomer of chiral substrates, which is highly dependent on the size of the two substituents attached to the phosphorus center and the dimensions of its substrate binding sites.^48−52^

Hitherto, several PTE variants have been generated where the stereoselectivity for different organophosphorus substrates has been increased, relaxed, or reversed via introduction of several amino acid substitutions in the three substrate binding sites.^49,52,69^ While the manipulation of stereoselectivity of PTE through mutations occurs at the sequence level and thus, cannot be reversed or undone after the protein has been expressed and purified, we attempted to achieve the photocontrol of stereoselectivity by applying light as an external stimulus. In the present work, we used GCE to incorporate ONBY within the large subsite of the active center of PTE to achieve control of stereoselectivity driven by a light stimulus. This proof of concept was done with substrates with a *p*-nitrophenol leaving group in combination with either methyl, ethyl, phenyl, or cyclohexyl substituents. Our results demonstrated that, depending on the incorporation of additional mutations within the small subsite and the specific nature of the substrate, significant alterations of enantioselectivity or even its complete reversal can be achieved by light-induced decaging of ONBY. Subsequent computational analysis revealed that the molecular mechanism underlying the observed stereoselectivity switch is based on a complex interplay between the steric constraints imposed by ONBY and its interactions with the substrate and the protein.

## Results and Discussion

### Design, Expression, and Purification of PTE Variants

**Figure 2:**
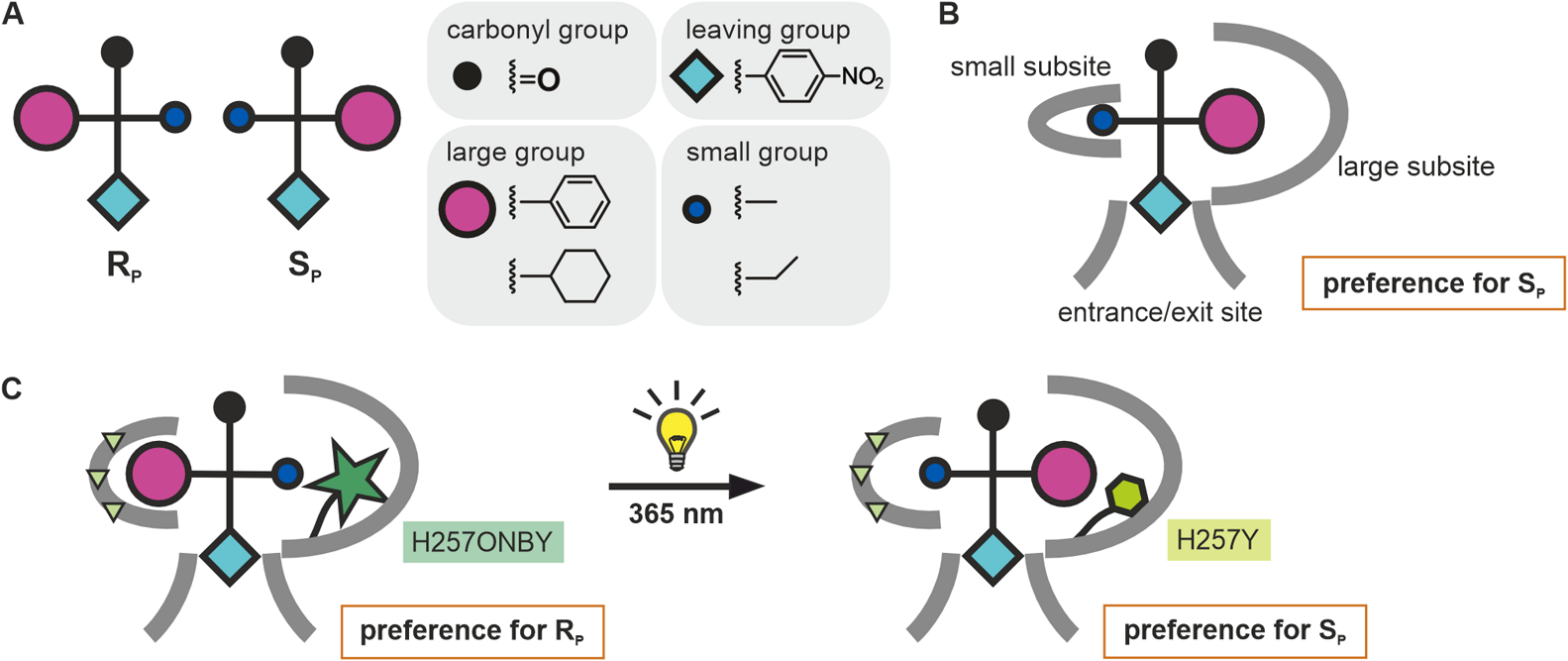
Initial design strategy for the photocontrol of PTE stereoselectivity. (A) Schematic representation of R_P_ and S_P_ enantiomers of chiral *p*-nitrophenyl substrates (Fischer projection). (B) Wild-type PTE preferentially hydrolyzes the respective S_P_ enantiomer, because the cyclohexyl or phenyl substituents are bound to the large subsite, while the small subsite is occupied by either the ethyl or methyl group. (C) Presumed photoinduced inversion of enantioselectivity via UV-induced decaging of incorporated ONBY. The exchange of His257 with ONBY in combination with small subsite enlarging mutations (indicated as yellow triangles) was deemed to result in preferential binding and hydrolysis of the R_P_ enantiomers. Following cleavage of ONBY by irradiation at 365 nm, the large subsite is re-enlarged, which should restore the preference for the S_P_ enantiomer.

The active center of PTE is composed of three subsites (Figure 1), of which the small and the large subsites are crucial for substrate orientation and thus, determine stereoselectivity. Accordingly, the substitution of certain amino acids that are associated with these subsites have previously been reported to affect the enantiomeric preference of PTE.^49−52,67^ Inspired by these findings, we aimed for a UAA-based approach to establish the artificial photocontrol of PTE stereoselectivity. The tested substrates are schematically displayed in Figure 2A and the composition of the active site in wild-type PTE, which was the starting point of our approach and leads to the preferential turnover of the S_P_ enantiomers, is displayed in Figure 2B. If the S_P_ enantiomer of the tested substrates is preferred, the larger substituent occupies the large subsite. We initially reasoned that the incorporation of the bulky ONBY into the large subsite would lead to the downsizing of this site, which might then no longer be voluminous enough to harbor the cyclohexyl or phenyl substituents of the substrate. Instead, the ethyl or methyl group of the substrate were assumed to bind to the ONBY-modified large subsite, accompanied by a shift of the stereoselectivity from the S_P_ to the R_P_ enantiomer (Figure 2C). We then reasoned that this situation could be further promoted by enlarging the small subsite via the substitution of voluminous amino acid residues with smaller ones, facilitating the binding of the cyclohexyl or phenyl moieties. Following UV-irradiation, the caging group would be removed to yield tyrosine, resulting in the re-enlargement of the large subsite. Hence, the preference for the S_P_ enantiomer would be restored and consequently, the light-induced switch in stereoselectivity from the R_P_ to the S_P_ enantiomer would be achieved.

The large subsite residue His257 was selected to be replaced by ONBY, because decaging would lead to a His to Tyr exchange, which has previously been shown to be compatible with a high catalytic activity of PTE.^52^ In addition to the PTE variant H257ONBY, the small subsite was systematically enlarged by mutation of Ile106, Phe132, and Leu303 to alanine as well as His254 to glycine, either individually or in certain combinations. As a control, variants containing tyrosine instead of ONBY at position 257 were generated and analyzed as well.

The PTE gene from *P. diminuta* encodes an N-terminal leader peptide of 29 amino acids. Since it has been shown to have a negative effect on activity, all PTE variants were produced without this leader peptide.^70^ The variants were generated by heterologous gene expression in *Escherichia coli* and purified from the soluble cell extract by immobilized metal ion affinity chromatography, followed by buffer exchange either via dialysis or via preparative size exclusion chromatography. Proteins containing ONBY were coexpressed with the corresponding tRNA^CUA^/aminoacyl-tRNA synthetase pair from *Methanocaldococcus jannaschii* that has been optimized for the binding of ONBY.^71^ The PTE variants were obtained with purities >90% (Figure S1) and with yields between 1.2 and 177 mg per liter expression culture (Table S1). In general, proteins consisting only of natural amino acids displayed overall very high yields compared to ONBY-containing ones that displayed low to moderate yields. The lower yields of proteins with incorporated UAAs are in accordance with previous findings.^16,17^

Since the aminoacyl-tRNA synthetase evolved for GCE with ONBY is based on a natural tyrosyl-tRNA synthetase from *M. jannaschii*,^71^ some misincorporation of tyrosine at the target site might occur during translation. In addition, previous studies have shown that during protein expression in *E. coli* cells a fraction of ONBY may be irreversibly reduced, resulting in *o*-aminobenzyl tyrosine (OABY), which can no longer be decaged to tyrosine.^20,72^ Having this in mind, targeted mass spectrometry experiments (selected reaction monitoring, SRM) were performed with all ONBY-variants to determine the fractions of ONBY, OABY, and tyrosine at position 257 before (as isolated = a.i.) and after irradiation (Table S2, Figure S2). All variants showed a high degree of ONBY, reflecting a well-functioning protein expression with minor amounts of misincorporation. After irradiation, a predominant fraction of ONBY was decaged resulting in an almost quantitative conversion to tyrosine. Remarkably, no OABY was detected in any of the PTE variants, indicating that ONBY was not reduced during expression in *E. coli*.

### Substrate Scope and Steady-State Activity Measurements

**Figure 3:**
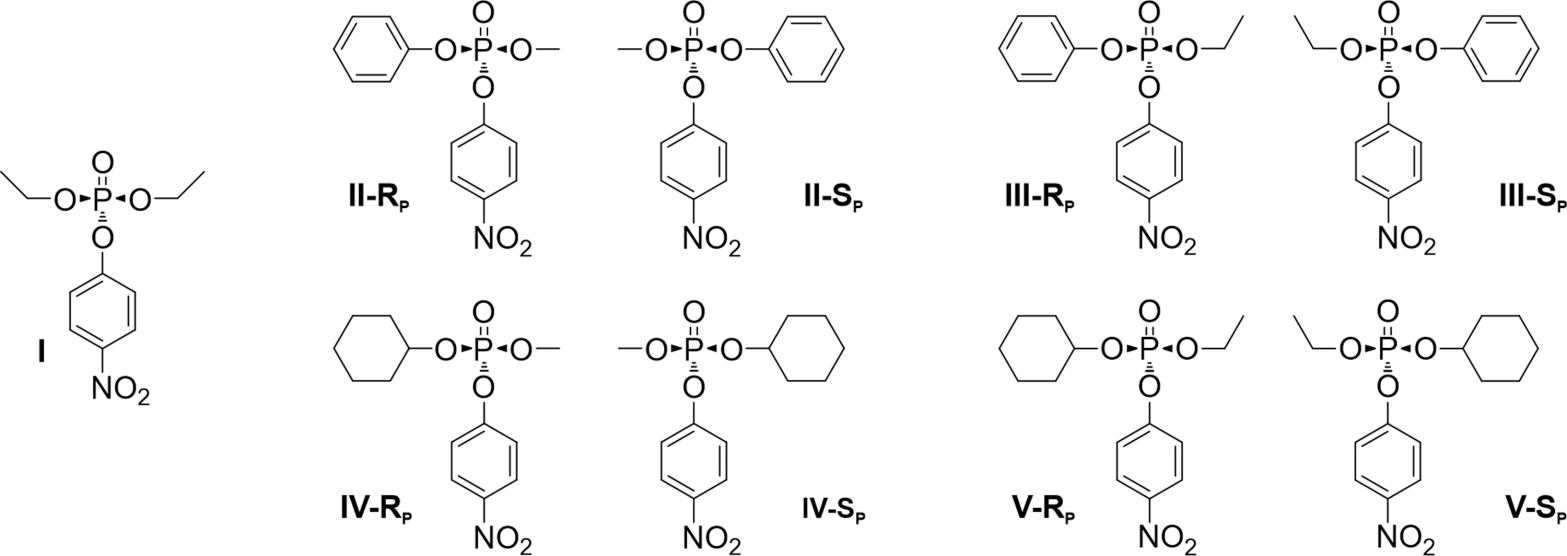
Substrate scope for PTE variants. The organophosphate triesters, which have a *p*-nitrophenyl leaving group, for which the PTE variants were tested are characterized by different substituents of the phosphoester. All substrates except compound I exist as racemic mixtures, where the size and the arrangement of the phosphoester substituents is decisive for the binding and catalytic turnover of each enantiomer.

To determine the effect of UV-irradiation on the enantioselectivity, the different PTE variants were assayed before and after irradiation with a series of organophosphate triester substrates containing *p*-nitrophenol as the leaving group. The whole substrate scope is depicted in Figure 3. Paraoxon (I) is the only analyzed substrate without a phosphorus chiral center and therefore, appears as only one stereoisomer. In contrast, substrates II–V exist as racemic mixtures consisting of the respective R_P_ and S_P_ enantiomers.

The release rate of *p*-nitrophenolate (Scheme 2) was monitored by absorbance spectroscopy under steady-state conditions. Assuming that the substrate concentration is much lower than the *K*_M_ value, the Michaelis–Menten equation can be rearranged in a manner that allows for the determination of the catalytic efficiency (*k*_cat_/*K*_M_) from a single hydrolysis curve (Equation S1), as performed in previous studies.^50,73^ To identify which enantiomer is preferentially hydrolyzed, a complementation assay was used by adding the strictly S_P_-selective variant PTE_G60A.^51^ If possible, the results of the complementation experiment were confirmed by ^31^P NMR analysis.

**Figure 4:**
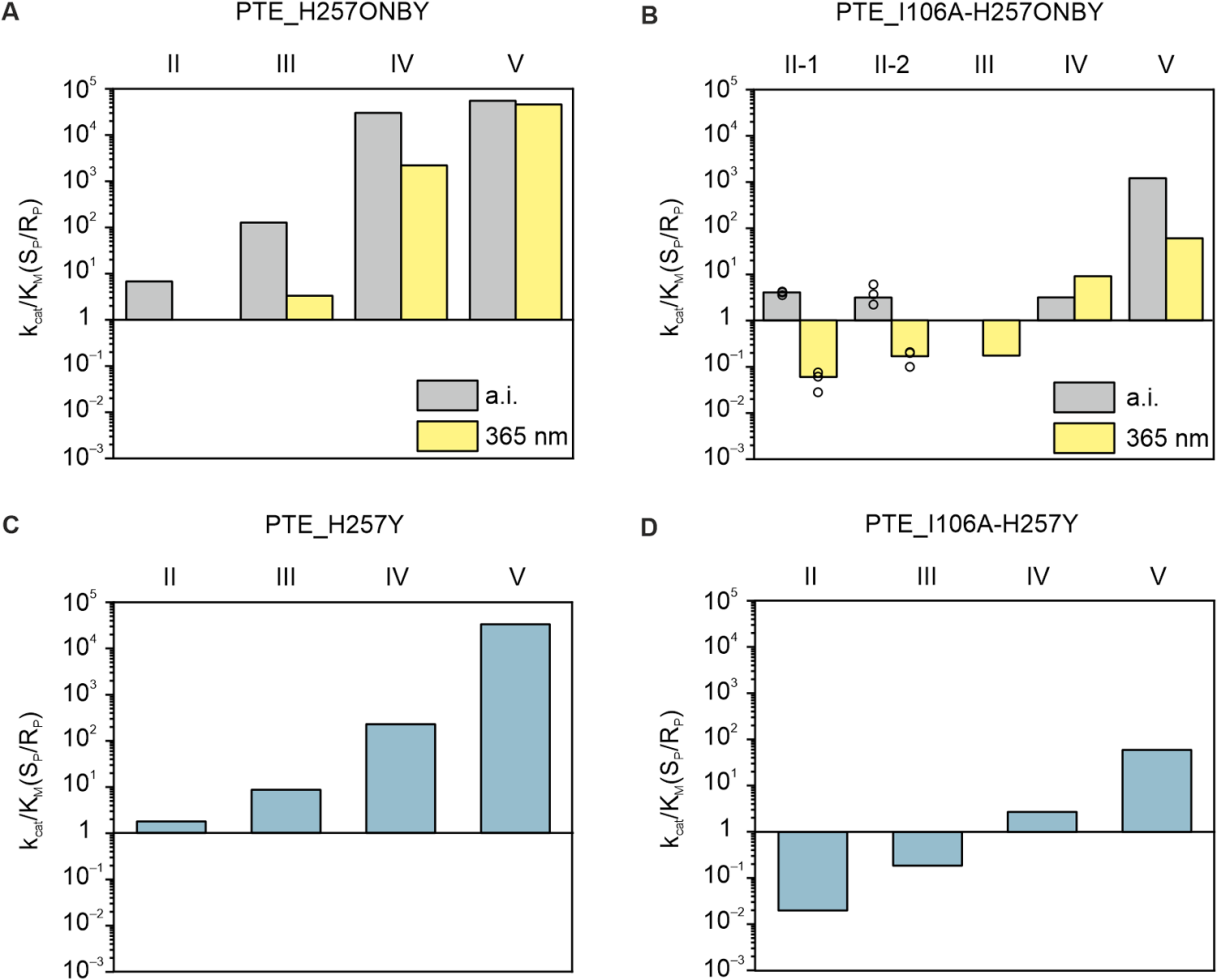
Stereoselectivities for the hydrolysis of chiral substrates by PTE_H257ONBY (A) and PTE_I106A-H257ONBY (B) as well as for the respective control variants PTE_H257Y (C) and PTE_I106A-H257Y (D). The enantioselectivities *k*_cat_/*K*_M_(S_P_/R_P_) before decaging (a.i.) and after decaging (365 nm) were calculated from the respective *k*_cat_/*K*_M_ values listed in Table S3. Biological duplicates of PTE_I106A_H257ONBY were assayed for hydrolysis of substrate II. The bar represents the mean of technical triplicates, and the corresponding single values are depicted as circles. Detailed enzyme and substrate concentrations are listed in Table S6.

**Figure 5:**
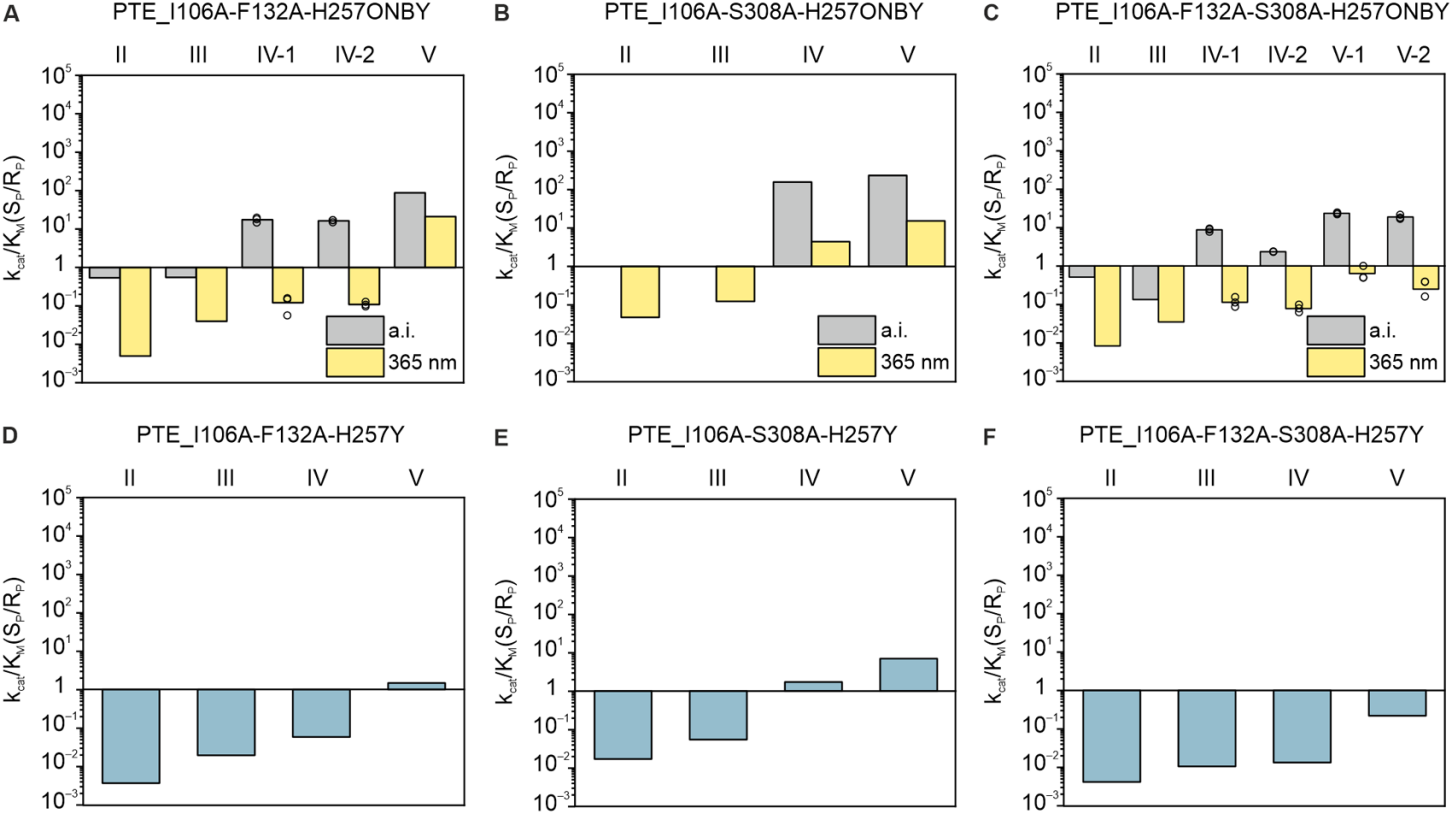
Stereoselectivities for the hydrolysis of chiral substrates by PTE_I106A-F132A-H257ONBY (A), PTE_I106A-S308A-H257ONBY (B), and PTE_I106A-F132A-S308A-H257ONBY (C), as well as for the respective control variants PTE_I106A-F132A-H257Y (D) and PTE_I106A-S308A-H257Y (E) and PTE_I106A-F132A-S308A-H257Y (F). The enantioselectivities *k*_cat_/*K*_M_(S_P_/R_P_) before decaging (a.i.) and after decaging (365 nm) were calculated from the respective *k*_cat_/*K*_M_ values listed in Table S3. Biological duplicates of PTE_I106A-F132A-H257ONBY were assayed for hydrolysis of substrate IV as well as of PTE_I106A-F132A-S308A-H257ONBY for substrates IV and V. The bar represents the mean of technical triplicates, and the corresponding single values are depicted as circles. Detailed enzyme and substrate concentrations are listed in Table S6.

The catalytic efficiencies of all PTE variants (including the tyrosine controls) for substrate I, as well as for both the S_P_ and R_P_ enantiomers for substrates II–V are shown in Table S3. The stereoselectivity of each variant for each pair of enantiomers is reported as the ratio of *k*_cat_/*K*_M_ for the S_P_ enantiomer relative to that for the R_P_ enantiomer [*k*_cat_/*K*_M_(S_P_/R_P_)] (Table S4). Hence, a stronger preference of the S_P_ enantiomer is reflected in a higher stereoselectivity. For the sake of clarity, enantioselectivities are shown as bar graphs in Figures 4, 5, and S3.

First, PTE wild-type (PTE_wt) and the S_P_-selective variant PTE_G60A were assayed with substrates I–V as control experiments. The enantioselectivities of PTE_wt and PTE_G60A obtained for the hydrolysis of the racemic substrates II and III are in good agreement with previously reported values and are more pronounced for substrates with bulkier substituents such as present in IV and V (Figure S3).^51,52^ This might be attributed to steric reasons, since the phenyl-moiety (II and III) adopts a planar conformation while the cyclohexyl-group (IV and V) engages a bulkier chair conformation, thus shifting the preference to hydrolysis of the S_P_ enantiomers of substrates IV and V. In accordance with literature, this effect is even more pronounced in PTE_G60A, where the increased steric constraints in the small subsite have been shown to hinder the turnover of the R_P_ enantiomer.^51,52^

### Photocontrol of Enantioselectivity

Prior to experiments to achieve photocontrol of enantioselectivity of racemic substrates, PTE_wt was subjected to irradiation with 365 nm and subsequently tested for the turnover of all substrates. The catalytic efficiencies (Table S3) as well as the resulting enantioselectivities (Table S4) are in good agreement those observed for the nonirradiated enzyme, confirming that exposure to light generally does not affect PTE activity. Furthermore, all variants containing ONBY and the corresponding controls were tested for the turnover of paraoxon (I) to ensure proper functionality. The results yielded *k*_cat_/*K*_M_ values between 1.7 × 10^5^ and 4.2 × 10^7^ M^–1^ s^–1^, validating high catalytic activities in all cases, both before and after irradiation (Table S3). Moreover, these results constitute an important control to demonstrate that the variants are not damaged by the applied irradiation conditions.

In a first attempt to achieve a light-induced change in stereoselectivity, PTE_H257ONBY was assayed for the hydrolysis of the racemic substrates II–V before and after irradiation at 365 nm. For substrates II and III, before decaging the S_P_ enantiomer was preferentially hydrolyzed, resulting in enantioselectivities of 8.6 (II) and 130 (III) (Table S4, Figure 4A). Following irradiation, a relaxation of stereoselectivity was observed for both substrates, mainly caused by an increased hydrolysis rate of the R_P_ enantiomer. For substrates IV and V, before decaging the hydrolysis rate for the S_P_ enantiomers was remarkably high, in contrast to a very low hydrolysis rate for the respective R_P_ enantiomers, resulting in enantioselectivities of 30000 (IV) and 55000 (V). Following irradiation, the stereoselectivities remained similar. For the control variant PTE_H257Y the enantioselectivities for substrates II–V agreed well with those of PTE_H257ONBY after decaging (Figure 4C). This implies that ONBY is quantitatively decaged after irradiation, which coincides with the results obtained from mass spectrometry experiments (Table S2).

Our initial design strategy anticipated that incorporation of ONBY might result in the preference of the R_P_ enantiomer. After irradiation, the preference of the S_P_ enantiomer should be restored since the H257Y mutation has previously been reported to result in a preference of the S_P_ enantiomer of substrates II and III^52^ (cf. Figure 2). However, the results obtained with PTE_H257ONBY showed that the S_P_ enantiomer was favored before irradiation and that decaging led to a promoted hydrolysis of R_P_ instead of the envisioned promotion of S_P_ and hence, relaxed rather than increased enantioselectivity. Based on these findings, we adapted our strategy and tried to achieve the photocontrolled inversion of stereoselectivity from S_P_ to R_P_ (instead of from R_P_ to S_P_). To this end, the hydrolysis rate of the R_P_ enantiomer should be further increased by enlarging the small subsite.

The first analyzed variant was PTE_I106A-H257ONBY. For substrate II, before decaging the S_P_ enantiomer was preferentially hydrolyzed, resulting in enantioselectivities of 4.0 and 3.1 in two biological replicates (#1 and #2) (Table S4, Figure 4B). Following irradiation, stereoselectivity was inversed to 0.06 (#1) and 0.17 (#2), corresponding to a 17-fold (#1) and a 6-fold (#2) preference for the R_P_ enantiomer. The 4-fold preference for S_P_ before and the 17-fold preference for R_P_ after irradiation translate into a significant stereoselectivity switch by a factor of 68. Importantly, this factor constitutes the lower limit for PTE_I106A-H257ONBY, considering that around 10% of tyrosine were already present before irradiation and 2% ONBY remained after decaging (Table S2). Exemplary hydrolysis curves of the complementation assay that testify to this stereoselectivity inversion are shown in Figure S4. The stereoselectivity switch was due to an increase of the rate of R_P_ hydrolysis while the rate for S_P_ hydrolysis remained identical before and after irradiation (Table S3). These results confirm the desired photoinduced inversion of stereoselectivity as anticipated in the adapted design strategy, wherein the reduction of the overall enantioselectivity facilitated the switch from the preferential hydrolysis of S_P_ to R_P_ after irradiation. For substrate III, before decaging both enantiomers were hydrolyzed at the same rate, corresponding to an enantioselectivity of 1. After decaging, the R_P_ enantiomer was preferentially hydrolyzed, resulting in an enantioselectivity of 0.17. For substrates IV and V, PTE_I106A-H257ONBY mainly preferred the hydrolysis of the S_P_ enantiomers with stereoselectivities of 3.1 (IV) and 1200 (V) before irradiation and 9.0 (IV) and 60 (V) after irradiation. For the control variant PTE_I106A-H257Y the enantioselectivities for substrates II–V were consistent with those of PTE_I106A-H257ONBY after decaging (Figure 4D), suggesting that the cleavage of ONBY to recover tyrosine at this position is nearly quantitative. Again, these findings are in accordance with the results obtained from mass spectrometry experiments (Table S2).

For substrates IV and V comprising a cyclohexyl instead of a phenyl substituent, the enantioselectivity after irradiation remains relatively high. To accomplish the stereoselective switch for these substrates, further enhancing the hydrolysis rate for R_P_ was deemed promising. Similar to I106A, the small subsite- and entrance/exit subsite-enlarging mutations S308A and F132A have previously been shown to significantly enhance the rate of hydrolysis for R_P_ enantiomers for a number of PTE substrates.^51^ The corresponding variants PTE_I106A-F132A-H257ONBY and PTE_I106A-S308A-H257ONBY were first assayed for substrates II and III and exhibited enantioselectivities close to 1 before decaging (Table S4, Figure 5A,B), while after irradiation clear preferences for the hydrolysis of the respective R_P_ enantiomers were observed, resulting in enantioselectivities of 5.0 × 10^–3^ (II) and 4.0 × 10^–2^ (III) for PTE_I106A-F132A-H257ONBY and of 4.8 × 10^–2^ (II) and 0.12 (III) for PTE_I106A-S308A-H257ONBY. For substrate IV, the two variants behaved differently. PTE_I106A-F132A-H257ONBY showed a preferential hydrolysis of the S_P_ enantiomer before decaging, resulting in enantioselectivities of 17 (#1) and 16 (#2) in two biological replicates (Table S4, Figure 5A). Following irradiation, stereoselectivity was inversed to 7.9 × 10^–2^ (#1) and 0.11 (#2), corresponding to a 13-fold (#1) and a 9-fold (#2) preference for the R_P_ enantiomer. The 17-fold preference for S_P_ before and the 13-fold preference for R_P_ after irradiation translate into an overall remarkable stereoselectivity inversion by a factor of 220, which is even higher than the one observed for PTE_I106A-H257ONBY and substrate II. Again, this factor constitutes the lower limit for PTE_I106A-F132A-H257ONBY, considering that around 4% of tyrosine were already present before irradiation (Table S2). Exemplary hydrolysis curves of the complementation assay demonstrating this stereoselectivity switch are shown in Figure S5A,B. Moreover, the enantiomers of substrate IV gave resolvable signals in NMR measurements, which allowed for a further validation of the preferentially hydrolyzed enantiomer before and after irradiation (Figure S5C–E). Hence, as observed for PTE_I106A-H257ONBY with substrate II, the results obtained with PTE_I106A-F132A-H257ONBY with substrate IV revealed an inversion of stereoselectivity from a preferential hydrolysis of S_P_ to R_P_ after irradiation. PTE_I106A-S308A-H257ONBY also showed a preferential hydrolysis of the S_P_ enantiomer before decaging, yielding an enantioselectivity of 1.6 × 10^2^, which was relaxed to 4.4 following irradiation (Table S4, Figure 5B). As observed for substrates II and III, for substrate V the two variants behaved similarly. Both favored the S_P_ enantiomer before decaging, yielding enantioselectivities of 87 for PTE_I106A-F132A-H257ONBY and 230 for PTE_I106A-S308A-H257ONBY while after irradiation stereoselectivity was relaxed to 21 and 15, respectively. For the control variants PTE_I106A-F132A-H257Y and PTE_I106A-S308A-H257Y the enantiomer preferences for substrates II–V coincided well with those of PTE_I106A-F132A-H257ONBY and PTE_I106A-S308A-H257ONBY after decaging (Figure 5D,E). Again, this implies that cleavage of ONBY to recover tyrosine is basically quantitative, which is consistent with the results obtained from mass spectrometry experiments (Table S2).

To enlarge the small subsite even more, all three substitutions were combined, yielding PTE_I106A-F132A-S308A-H257ONBY. For substrates II and III, before decaging the R_P_ enantiomer was preferentially hydrolyzed, resulting in enantioselectivities of 0.51 (II) and 0.13 (III), which were further decreased to 8.4 × 10^–3^ (II) and 3.5 × 10^–2^ (III) after irradiation (Table S4, Figure 5C). For substrate IV, PTE_I106A-F132A-S308A-H257ONBY showed a preferential hydrolysis of the S_P_ enantiomer before decaging, resulting in enantioselectivities of 8.7 (#1) and 2.3 (#2) in two biological replicates. Following irradiation, stereoselectivity was inversed to 0.12 (#1) and 7.8 × 10^–2^ (#2), corresponding to an 8.3-fold (#1) and a 13-fold (#2) preference for R_P_. The 8.7-fold preference for the S_P_ enantiomer before and the 8.3-fold preference for the R_P_ enantiomer after irradiation translate into a significant stereoselectivity switch by a factor of 72. This factor constitutes the lower limit for PTE_I106A-F132A-S308A-H257ONBY, considering that around 4% of tyrosine were already present before irradiation and 2% ONBY remained after decaging (Table S2). Exemplary hydrolysis curves from the complementation assay demonstrating this stereoselectivity switch are shown in Figure S6A,B. Moreover, NMR allowed for a further validation of the preferentially hydrolyzed enantiomers before and after irradiation (Figure S6C–E). Similar results were observed for substrate V where PTE_I106A-F132A-S308A-H257ONBY preferentially hydrolyzed the S_P_ enantiomer before decaging, resulting in enantioselectivities of 23 (#1) and 19 (#2) in two biological replicates (Table S4, Figure 5C). Following irradiation, stereoselectivity was inversed to 0.62 (#1) and 0.25 (#2), corresponding to a 1.6-fold (#1) and a 4-fold (#2) preference for the R_P_ enantiomer. The 19-fold preference for the S_P_ enantiomer before and the 4-fold preference for the R_P_ enantiomer after irradiation translate into a stereoselectivity switch by a factor of 76. This factor constitutes the lower limit for PTE_I106A-F132A-S308A-H257ONBY, considering that around 4% of tyrosine were already present before irradiation (Table S2). Exemplary hydrolysis curves from the complementation assay demonstrating this stereoselectivity switch are shown in Figure S7A, B. Moreover, like for substrate IV, the enantiomers of substrate V gave resolvable NMR signals, which allowed for a further validation of the preferentially hydrolyzed enantiomers before and after irradiation (Figure S7C–E). For the control variant PTE_I106A-F132A-S308A-H257Y the enantioselectivities for substrates II–V agreed well with those of PTE_I106A-F132A-S308A-H257ONBY after decaging (Figure 5F), indicating the quantitative decaging of ONBY as confirmed by MS/MS analysis. Hence, similarly to the observation made for PTE_I106A-H257ONBY with substrate II and for PTE_I106A-F132A-H257ONBY with substrate IV, also the results for PTE_I106A-F132A-S308A-H257ONBY with substrates IV and V demonstrate an inversion of stereoselectivity from a preferential hydrolysis of S_P_ to R_P_.

### Computational Structural Analysis of the Stereoselectivity Switch

The introduction of ONBY in combination with further mutations and its subsequent decaging resulted for some variants in the switch of enantiomer preference from S_P_ to R_P_. This is the opposite effect compared to what was expected based on the initial structural considerations for the design strategy (cf. Figure 2). To revise the structural rationale of the initial design strategy we performed a deeper computational analysis of the possible impact of ONBY on the active site architecture. Whereas the initial design strategy solely considered steric constraints within the binding pocket composition, our adapted approach also included possible stabilizing interactions of ONBY with the substrate and conformational changes of the active site induced by ONBY. In this context, we generated structural models for the active sites of PTE_I106A-H257ONBY with bound substrate II (representative of substrates with a phenyl substituent) and PTE_I106A-F132A-H257ONBY with bound substrate IV (representative of substrates with a cyclohexyl substituent). For comparison, the active sites of the respective PTE_I106A-H257Y and PTE_I106A-F132A-H257Y control variants were also modeled. The general modeling procedure is described in the following and methodological details are provided in the computational modeling part of the Material and Methods section. All structural models were generated with both, the S_P_ and the R_P_ enantiomer. To begin with, we used the PTE crystal structure (PDB: 1dpm) with the bound inhibitor diethyl 4-methylbenzylphosphonate.^64^ The inhibitor was replaced by an active orientation of the respective substrate and the two zinc ions were replaced by cobalt ions to match the experimental setup (Figure S8). To obtain the energetically preferred active site architectures for the variants with ONBY and the control variants, a stepwise rotamer search of the respective mutated residues including energy optimization of the active site with bound substrate was performed. Importantly, all four predicted active site architectures showed that, in principle, binding of the respective substrates is possible. However, no significant differences in the binding of the R_P_ and S_P_ enantiomer within the given methodological accuracy were observed (Figure S9).

**Figure 6:**
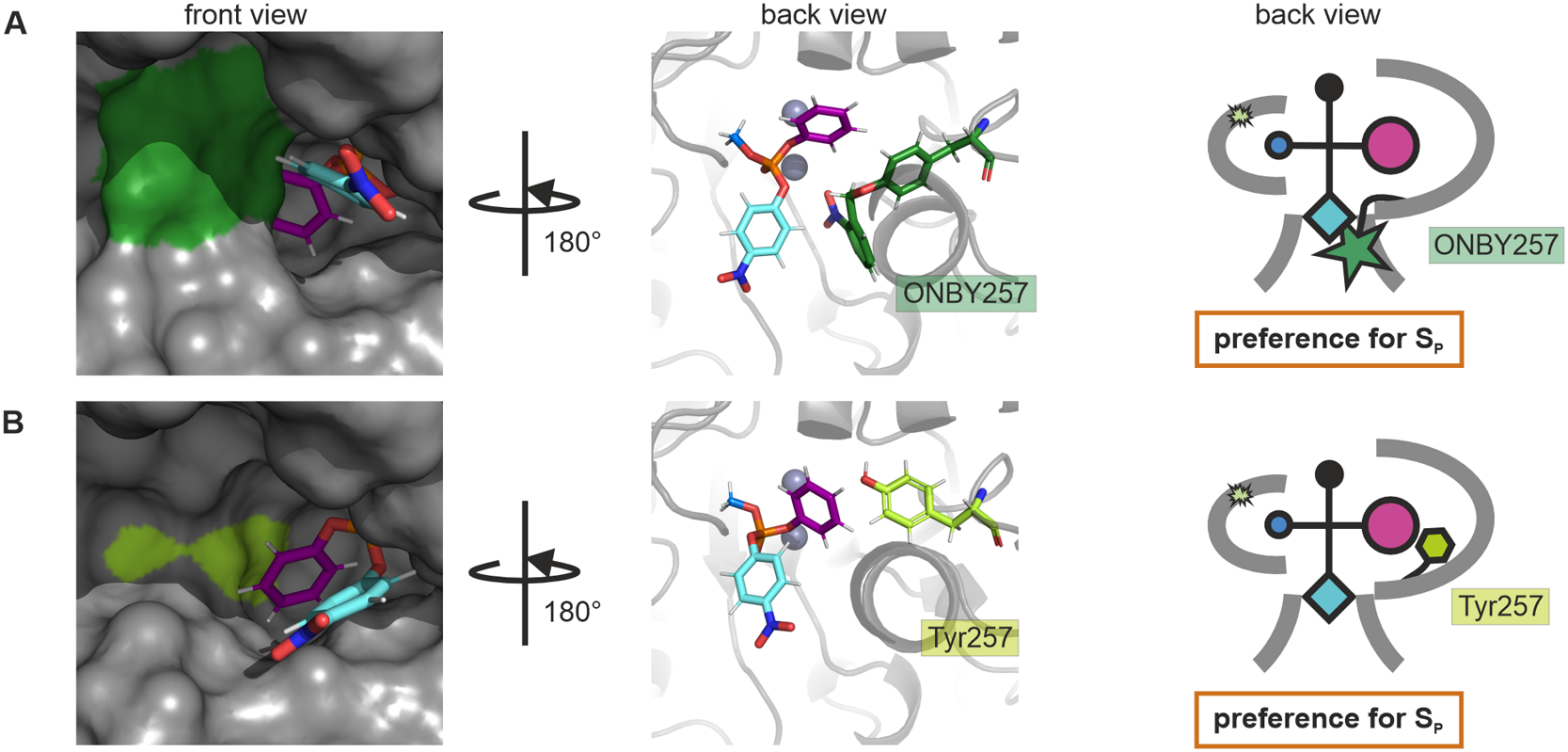
Revised structural rationale for the photocontrol of PTE stereoselectivity. Structural models of the active sites of PTE_I106A-H257ONBY (A) and PTE_I106A-H257Y (B) with the bound S_P_ enantiomer of substrate II. Color coding is according to Figure 2. Left panel: Surface representation of the front view of the active site structural model. Middle panel: Carton and sticks representation of the back view of the active site structural model. Right panel: Schematic representation deduced from the predicted structural models. Co^2+^ ions are depicted as gray spheres.

The results for PTE_I106A-H257ONBY are shown in more detail in Figure 6. Surprisingly, the tyrosine moieties of ONBY257 and Tyr257 occupied about the same space within the large subsite. However, the *o*-nitrobenzyl group of ONBY257 was not oriented in the large subsite, in contrast to the initial assumption (cf. Figure 2) but was rather rotated to the entrance/exit site (Figure 6A). Consequently, ONBY257 narrowed the entrance/exit site and not the large subsite, explaining why we did not observe the expected preference for the R_P_ enantiomer of the ONBY variants before decaging. Hence, when just considering the proportions of the large subsite, as done in the initial design, neither the introduction of ONBY nor its decaging has an influence on the occupied volume (Figure 6B). This is in accordance with the similar enantiomer preferences observed for PTE_H257ONBY before and after decaging (Table S4, Figure 4A), implying that the stereoselectivity switch observed for PTE_I106A-H257ONBY, PTE_I106A-F132A-H257ONBY, and PTE_I106A-F132A-S308A-H257ONBY may be caused by a different mechanism than the influence of ONBY in the large subsite.

In the search for such a mechanism, it was interesting to observe that based on its location in the predicted structural model, ONBY257 could interact in a similar manner as Tyr257 with the large substituent of the substrate (Figure S9). In addition, the *o*-nitrobenzyl caging group of ONBY has the potential to also interact with the leaving group of the substrate by π-stacking in the entrance/exit site and the imidazole side chain of His201, which is involved in metal ion coordination. Presumably, interaction with His201 could impact the charge distribution and thus, the overall active site architecture, thereby influencing the hydrolysis reaction. Furthermore, interaction with the substrate leaving group may affect substrate positioning, thereby altering enantiomer selectivity, and narrowing the entrance/exit site may have an impact on the substrate binding and leaving rate. These possible interactions and the associated effects would then be abolished by decaging and removal of the *o*-nitrobenzyl group. However, since the actual active conformation of the PTE active site remains elusive, the contribution of these potential interactions to alter the hydrolysis rates of the S_P_ and R_P_ enantiomers toward a stereoselective switch are highly challenging to predict and would require more in-depth computational analyses.

Computational analysis thus revealed a much more complex effect on the active site architecture and charge distribution by incorporation and decaging of ONBY in the respective variants than anticipated in the initial simplistic design model, thereby providing preliminary explanations for the discrepancy of the experimental results (stereoselectivity switch from a preferred conversion of S_P_ before irradiation to a preferred conversion of R_P_ after irradiation, rather than vice versa). The observed effect of the incorporated ONBY on the entrance/exit side indicates that besides the substrate bound state also the association and dissociation pathways impact enantioselectivity. However, a more comprehensive hypothesis for the molecular mechanism of this stereoselectivity switch upon decaging of ONBY must await a much more sophisticated computational study including quantum chemical calculations to reflect the impact on the charge distribution and simulations of the dynamics during substrate entry and release.

## Conclusion

PTE is an enzyme that is capable of hydrolyzing a wide variety of organophosphorus substrates including compounds that are severely toxic. Most of these compounds exist as racemic mixtures where PTE typically prefers one enantiomer over the other, governed by the stereochemical constraints in the three binding subsites. In this work, going beyond the possibilities that substitutions with natural amino acids provide, we combined a rational design strategy with the integration of the light-sensitive UAA ONBY to accomplish the light-induced photocontrol, more precisely the inversion of enantioselectivity of PTE and could achieve a switch in stereoselectivity for three of the four racemic substrates tested. The best variant exhibited a 220-fold switch in stereoselectivity upon irradiation which shows that the goal of this work has been fully achieved. Bioinformatic analysis showed that ONBY does not contribute to the reshaping of the large subsites as expected which substantiates the fact that incorporation of UAAs and their mode of action are hardly predictable and thus, highlights the importance of sophisticated *in silico* methods for the validation of experiments with UAAs. Taken together, our findings significantly expand the repertoire of possibilities for regulating enantioselective transformations in biocatalytic systems and at the same time provide an elegant solution for their remote, spatiotemporal control.

## Materials and Methods

### Chemicals and Substrates

ONBY and Paraoxon (I) were purchased from Sigma-Aldrich (>95% pure). Compounds II, III, IV, and V were synthesized as described previously.^58,74,75^ All other chemicals were purchased from commercial sources and were of analytical grade or higher.

### Subcloning and Site-Directed Mutagenesis

The gene encoding PTE except the N-terminal leader peptide (amino acids 1–29) was codon-optimized for the expression in *E. coli* and synthesized by ThermoFisher Scientific with BsaI restriction sites. The gene string was cloned into pET21a_BsaI applying GoldenGate cloning^76^ and integrity of the coding region was confirmed by Sanger Sequencing. For site-directed mutagenesis of PTE, a modified version of the QuickChange protocol^77^ was applied. Primers containing the desired mutations (Table S5) were designed to amplify the entire target plasmid and the resulting linear amplicon was subsequently treated with 5 U T4 Polynucleotide Kinase and 10 U T4 DNA Ligase (ThermoFisher Scientific) in 1x T4 Ligase Buffer at 37 °C for 30 min, followed by incubation at room temperature for another 30 min. The presence of the desired mutations was confirmed by Sanger Sequencing.

For cloning of pEVOL_ONBY-RS, a pEVOL plasmid provided by Peter Schultz (Scripps Research Institute, La Jolla, Ca, USA) served as plasmid backbone. The ONBY-RS gene^71^ was codon optimized for *E. coli* and synthesized by ThermoFisher Scientific with BsaI restriction sites. Since the pEVOL plasmid typically harbors two copies of synthetases, cloning of ONBY-RS was performed in two steps. In the first step, the pEVOL backbone was prepared with suitable primers for site 1 (Table S5), while in the second step suitable primers for site 2 (Table S5) were used before GoldenGate cloning with BsaI was employed.^76^ The integrity of the entire plasmid was confirmed by Sanger sequencing.

### Protein Production and Purification

PTE_wt and variants were produced by heterologous gene expression in *E. coli* BL21 (DE3) gold (Agilent Technologies) with a C-terminal His_6_-tag. All proteins were expressed and purified in the presence of CoCl_2_ since the Co^2+^-bound form of the enzyme shows a higher activity compared with other divalent cations.^65^ The cells were transformed with the respective expression vectors and grown in LB medium supplemented with ampicillin (150 mg/mL) at 37 °C to an OD_600_ of 2–4. Gene expression was induced by addition of 0.5 mM isopropyl-β-thiogalactopyranoside (IPTG) and cells were further incubated at 25 °C overnight. Cells were harvested by centrifugation and suspended in 50 mM Tris/HCl pH 8.5, 150 mM NaCl, 0.1 mM CoCl_2_, and 10 mM imidazole. After cell lysis by sonification (Branson Sonifier W-250D; amplitude 50%; 2 min, 30 s pulse/30 s pause), the debris was removed by centrifugation. Proteins were purified from the supernatant using immobilized Ni^2+^ ion affinity chromatography (HisTrap FF Crude 5 mL, GE Healtcare) in 50 mM Tris/HCl pH 8.5, 150 mM NaCl, 0.1 mM CoCl_2_, 10 mM imidazole by applying a linear imidazole gradient (0–75%). Fractions containing the proteins were identified by SDS-PAGE analysis, pooled, and the buffer was exchanged to 50 mM Tris/HCl pH 8.5, 50 mM NaCl, either by preparative size exclusion chromatography at 4 °C, using a HiLoad 26/600 Superdex 200 PG column (GE Healthcare) or by dialysis at 4 °C overnight. In the case of later use, the proteins were concentrated and dripped into liquid nitrogen for storage at −80 °C.

For PTE variants containing ONBY, the expression protocol was slightly adapted. Cells were cotransformed with the expression vector of the respective PTE variant and the auxiliary plasmid pEVOL_ONBY-RS and grown in LB medium (600 mL) supplemented with ampicillin (150 μg/mL) and chloramphenicol (30 μg/mL) at 37 *C*°. As soon as an OD_600_ of 2–4 was reached, gene expression was induced by the addition of 0.5 mM IPTG, 0.2 mM ONBY, and 0.02% l-arabinose and cells were further incubated at 25 °C overnight. Proteins were purified as described above, however, all following steps were conducted in the dark.

### Irradiation with UV Light

Irradiation of PTE variants prior mass spectrometry experiments and activity measurements was performed using a 365 nm high power LED (LED ENGIN LZ4–44UV00–000) at 20 V and 850 mA. The samples were placed directly in front of the LED and irradiated twice for 2 min with a cooling phase of 1 min in between.

### Mass Spectrometry for Targeted Quantification of Unnatural Amino Acid Containing Peptides

Sample preparation was carried out in the dark due to the light-sensitivity of the caged proteins. Proteins were separated on an SDS-gel, cut out and subjected to in-gel tryptic digestion, as described in Bhagat et al.^20^ Peptides were reconstituted in 20 μL of 1% formic acid and separated by reversed-phase chromatography. The LC-MS/MS system consisted of an UltiMate 3000 RSLCnano System (Thermo Scientific, Dreieich) coupled via a NanoSprayII source (SCIEX) to a QTRAP4500. Peptides were separated on an Acclaim Pepmap100 C18 nano column (75 μm i.d.x150 mm, Thermo Fisher) with a C18 Acclaim Pepmap100 preconcentration column (100 μm i.D.x20 mm, Thermo Fisher) in front. At a flow rate of 300 nL/min, a 60 min linear gradient of 4% to 40% acetonitrile in 0.1% formic acid was run.

A QTRAP4500 mass spectrometer was used for the targeted quantification of peptides containing unnatural amino acids. First, a spectral library comprising wild-type and mutant peptide spectra was built from several LC-MS/MS discovery runs (DDA, data dependent analysis) on the hybrid triple quadrupole/linear ion trap instrument QTRAP4500 (SCIEX). Database search was performed with a customized database comprising the *Brevundimonas diminuta* phosphotriesterase wild-type entry from UniProt, as well as sequences of the PTE variants (PTE_H257Y, PTE_I106A-H257Y, PTE_I106A-F132A-H257Y, PTE_I106A-S308A-H257Y, PTE_I106A-F132A-S308A-H257Y). In addition to general variable modifications as deamidation of asparagine and glutamine, oxidation of methionine, carbamidomethylation, and propionamide modification of cysteine, annotation of ONBY or OABY containing peptide species required setting +135.032 Da for *o*-nitrobenzyl-l-tyrosine (ONBY) and +105.058 Da for 2-aminobenzyl-l-tyrosine (OABY) as variable modifications. To create an SRM (Selected Reaction Monitoring) method for targeted quantification, the open source software Skyline (MacCoss Lab Software, Seattle, USA) was used. According to their occurrence in the DDA runs, precursor charge states +2, + 3 or +4, each with 4 transitions, were included in the targeted method and the resulting transition list was imported into the instrument software (Analyst 1.6.1). In addition, the following parameters were set for the SRM-method: Q1 and Q3 set to unit resolution (0.7 *m*/*z* half-maximum peak width), dwell time 20 ms, cycle time <3 s. Second, a scheduled SRM method was created in Skyline by annotating peptide retention times from the initial SRM run and setting the following parameters: cycle time: 2 s, retention time window: 5 min. The resulting wiff files of the SRM-measurements were imported into Skyline, which facilitated the quantification of the peak areas of the respective transitions. Relative quantification of the different peptide species was achieved by adding up peak areas of the nonmodified peptide species and the related ONBY and OABY containing peptide species. Assuming this sum to represent 100%, it was possible to calculate the percentage of the individual peptide species.

### Kinetic Measurements

All substrates used in this work comprise a *p*-nitrophenol group that is cleaved off by PTE. In principle, the production of *p*-nitrophenolate and thus, the reaction progress can be followed at 400 nm.^58^ However, small amounts of ONBY might accidentally be decaged when measuring at this wavelength since its absorption spectrum reaches up to 400 nm. To prevent unintentional cleavage of ONBY, the production of *p*-nitrophenolate was monitored at 420 nm, where absorption intensity was still high enough to unambiguously monitor the hydrolysis reaction. Initially, the concentration of each substrate was determined by following the time course of its hydrolysis with KOH (Figure S10). Kinetic measurements were performed with a JASCO V650 spectrophotometer at 25 °C. The reaction mixture contained 100 mM Tris/HCl pH 9.0, 0.1 mM CoCl_2_, 0.1 nM–20 μM PTE enzyme and 5–20 μM substrate (I–V) in a total volume of 3 mL. Enzymes were assayed either without irradiation (“as isolated” = a.i.) or preirradiated with UV light at 365 nm before the reaction was initiated by the addition of substrate. Substrate and enzyme concentrations were adapted for each measurement individually and are listed in Table S6. By applying 5–20 μM substrate, it was assured that the substrate concentration remained much lower than the Michaelis constant *K*_M_.^50^ Under those conditions, the time courses follow pseudo-first order kinetics and the Michaelis–Menten equation can be rearranged to allow for the determination of catalytic efficiencies *k*_cat_/*K*_M_ by fitting a single or double exponential function to the data (Equation S1). The single exponential fit was employed if the turnover of one enantiomer (S_P_ or R_P_) was monitored or if both enantiomers were degraded at approximately the same rate. The double exponential fit was used when both enantiomers were hydrolyzed at different rates. To identify which enantiomer is preferentially hydrolyzed, a complementation assay was used by adding the strictly S_P_-selective variant PTE_G60A (5–15 nM) as soon as one enantiomer was almost completely hydrolyzed.^51^ The enantioselectivity was calculated from the ratio of catalytic efficiencies [*k*_cat_/*K*_M_(S_P_)]/[*k*_cat_/*K*_M_(R_P_)]. Due to the large number of substrates and variants to be tested, all measurements were initially carried out only once. For variant-substrate combinations that showed an inversion of stereoselectivity after irradiation, biological duplicates were prepared in the form of two independent protein expressions and purifications. These were measured in technical replicates, three times each.

### ^31^P Nuclear Magnetic Resonance (NMR) Analysis of Enzymatic Reactions

^31^P NMR analysis was performed for the variants that showed an inversion of stereoselectivity for substrates IV and V after irradiation to additionally verify the results obtained from the complementation assays. While there were no differential resonances associated with the two enantiomers for substrates II and III, assignment of the enantiomers of substrates IV and V to two distinct resonances was possible. Reactions were conducted in a total volume of 50 mL in 100 mM Tris/HCl pH 9.0, with 200 μM substrate IV or V, and 0.1 mM CoCl_2_. The reaction was started by the addition of enzyme and quenched with DCM after approximately one-third of the racemic substrate was hydrolyzed. The aqueous phase was dried over Na_2_SO_4,_ and the solvent was removed under reduced pressure. The residual substrate was dissolved in 700 μL CDCl_3_ and 175 mg of the chiral shift reagent Fmoc-Trp(Boc)–OH were added. ^31^P NMR spectra were recorded using a Burker Avance 400 Spectrometer (400.13 MHz). The strictly S_P_-selective variant PTE_G60A (2 nM, reaction time: 2 min) was used to assign each peak to the respective enantiomer of both substrates IV and V. PTE_I106A-F132A-H257ONBY and PTE_I106A-F132A-S308A-H257ONBY were used either nonirradiated (as isolated = a.i.) or preirradiated with UV light. The exact enzyme concentrations, as well as the reaction durations, are given in the caption of the respective figures.

### Computational Modeling

To investigate the ligand-protein interactions and the role of ONBY on the active site architecture, structural models of different PTE variants with bound substrate were constructed. In detail, we generated structural models for the active sites of PTE_I106A-H257ONBY with bound substrate II and PTE_I106A-F132A-H257ONBY with bound substrate IV. For comparison, models of the respective PTE_I106A-H257Y and PTE_I106A-F132A-H257Y variants were constructed. All structures were modeled with both the S_P_ and the R_P_ enantiomer. The basis for the modeling was the crystal structure of PTE_wt with the bound inhibitor diethyl-4-methylbenzylphosphonat (PDB: 1dpm)^64^ and two zinc ions within the active site (Figure S8A). The inhibitor was replaced by an active orientation of the respective substrates as shown in Figure S8C. Therefore, the organic substituents of the substrates were aligned with the rests of the inhibitor. Furthermore, the two zinc ions were replaced by cobalt ions to match the experimental setup. To obtain the energetically preferred active site architecture for the PTE_H257ONBY and PTE_H257Y variants, we performed a stepwise rotamer search of the respective mutated residues including energy optimization of the active site with bound substrate. All possible side chain torsion angles were independently rotated in 5° steps and subsequently optimized to cover the full conformational space of the side chains. The finals result was the conformation with the minimal energy based on a force filed energy calculation. For energy optimization and scoring we utilized the AMBER force field^78^ and a Generalized Born solvent model^79^ implemented within the MAXIMOBY software package version 2024 (CHEOPS, Germany).

### Force Field Parameterization Strategy

To perform energy optimization and energy analysis, we first derived parameters for the original AMBER All Atom force field^78^ for the inhibitor diethyl-4-methylbenzylphosphonat as well as substrates II and IV, the cobalt ions, the carbonic acid that is bound to Lys169, and ONBY. We followed the parametrization strategy for the original AMBER All Atom force field.^78^ First, the partial charges are calculated by an ESP-fitted STO-3*G*/6-31G* single point charge calculation of the cobalt ions with their respective ligand sphere, utilizing GAUSSIAN09.^80^ To approach the difficult nature of the open-shelled ground state cobalt, the charge calculations were performed with zinc as a substitute ion.^81^ For a seamless integration into the AMBER force field, the charges of the tyrosine component of ONBY were retained with only the nitrobenzene being calculated and merged with tyrosine (Figure S11).

To check the adequate parametrization, we prepared and optimized the crystal structure of PTE_wt with bound inhibitor. Comparison of this structure with our optimized structure in Figures S8A and Figure S8B showed that the ion coordination sphere remains stable without significant differences. Thus, our derived parameters were validated.

### Computational Analysis Strategy

To identify the key interaction motives within the active sites we analyzed the intramolecular contact pattern utilizing the contact matrix algorithm in MAXIMOBY version 2024 (CHEOPS, Germany) and the PyContact plugin for VMD.^82^ Thereby, stabilizing hydrophilic and hydrophobic interactions between the protein, ions, and the respective substrates were identified. Additionally, we identified key protein–protein and protein-ion interactions stabilizing the shape of the active site and thereby promoting or interfering with the binding interface of the different substrates.

## Supporting Information Available

Additional experimental details, raw data of hydrolysis curves, NMR and MS experiments, as well as corresponding structural analyses (PDF)

## ABBREVIATIONS

PTE: phosphotriesterase (from *Pseudomonas diminuta*)
PDB: Protein Data Bank
GCE: genetic code expansion
UAA: unnatural amino acid
ONBY: *o*-nitrobenzyl-l-tyrosine
OABY: *o*-aminobenzyl tyrosine
a.i.: as isolated (nonirradiated protein sample)
