# Supplementary material for: Photocontrolling the Enantioselectivity of a Phosphotriesterase via Incorporation of a Light-Responsive Unnatural Amino Acid
Source: JACS Au. 2025 Feb 5;5(2):858–70. doi: 10.1021/jacsau.4c01106 (PMC11863162; doi:10.1021/jacsau.4c01106)
Supplement: Supplementary file 1 — au4c01106_si_001.pdf [file au4c01106_si_001.pdf]

Supporting Information

Photo-Controlling the Enantioselectivity of a Phosphotriesterase via Incorporation of a Light-Responsive Unnatural Amino Acid

Caroline Hiefinger[1&], Gabriel Zinner[1&], Torben F. Fürtges[1], Tamari Narindoshvili[2], Sebastian Schindler[1,2], Astrid Bruckmann[3], Till Rudack[1]*, Frank M. Raushel[2]*, Reinhard Sterner[1]*

[1]Institute of Biophysics and Physical Biochemistry, Regensburg Center for Biochemistry, University of Regensburg, D-93053 Regensburg, Germany

[2]Department of Chemistry, Texas A&M University, College Station, TX 77843-3255, USA

[3]Institute of Biochemistry, Genetics and Microbiology, Regensburg Center for Biochemistry, University of Regensburg, D-93053 Regensburg, Germany

[&] Equal contribution

*Corresponding authors:

till.rudack@ur.de; raushel@chem.tamu.edu; reinhard.sterner@ur.de

Table of Contents

S1. Supplemental Figures S3

S2. Supplemental Tables S13

S3. Supplemental Equations S21

S4. Supplemental References S22

Supporting Information

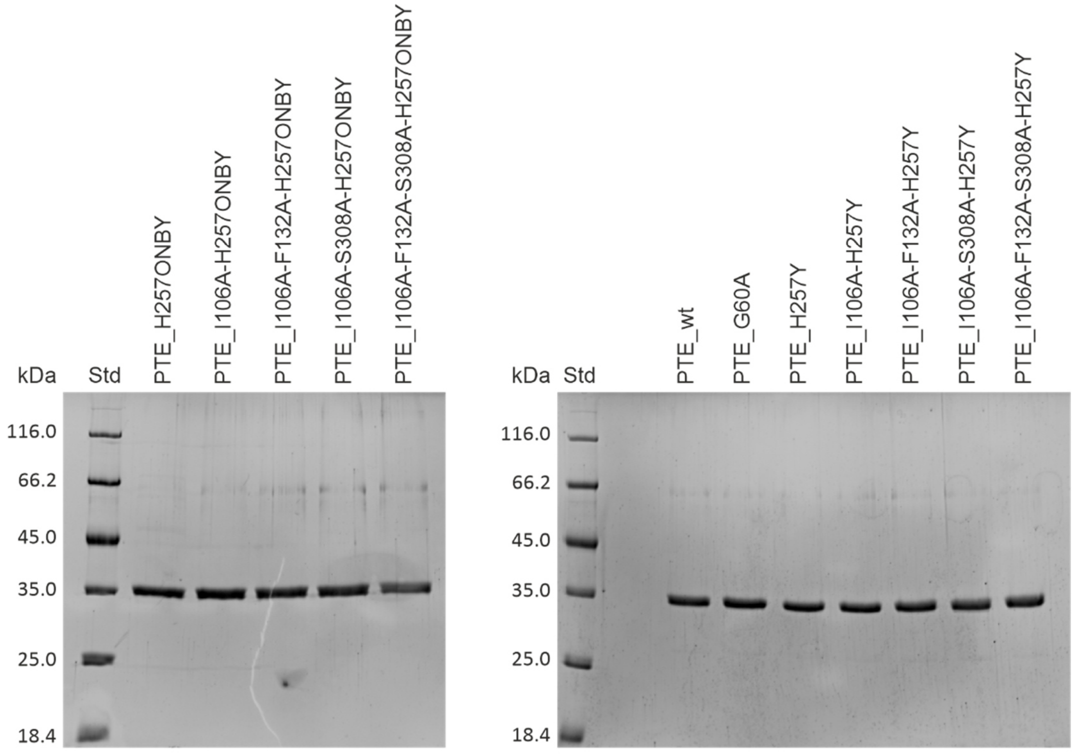

Figure S1. SDS-PAGE (13.5% acrylamide) of all PTE variants produced in this work. 7 µl low-molecular weight standard (Std) and 1 µg of each purified protein were applied to the gel.

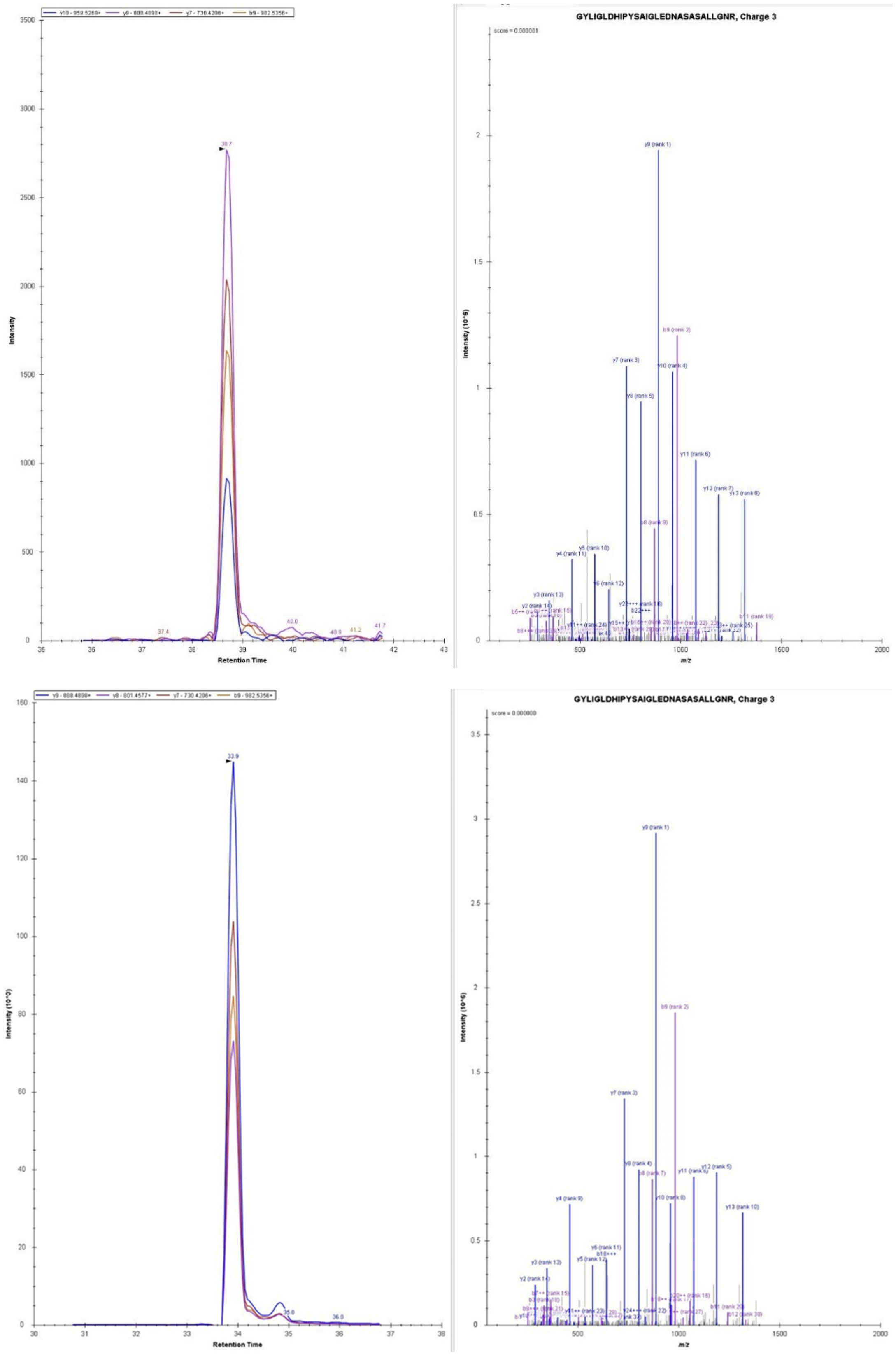

Figure S2. **Selected Reaction Monitoring (SRM) of caged and decaged H257ONBY peptide species.** Chromatographic traces of selected transitions of a caged H257ONBY peptide (upper left panel) and a decaged H257Y peptide (lower left panel) as well as corresponding MS/MS spectra of H257ONBY (upper right panel) and H257Y (lower right panel) from the spectral library.

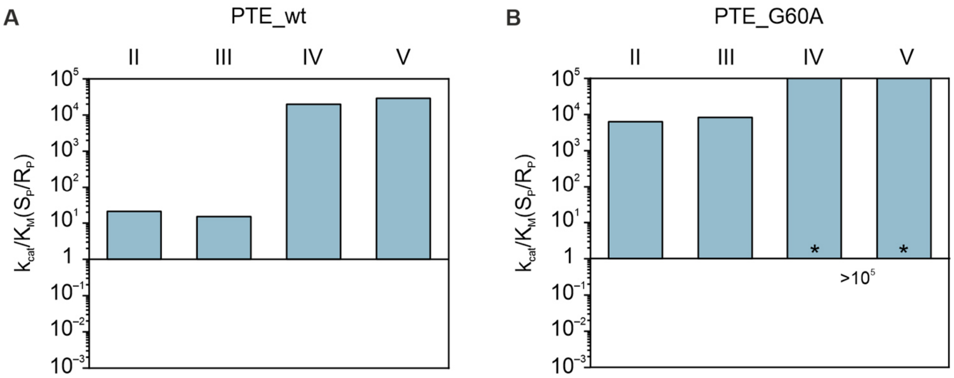

Figure S3. Stereoselectivities for the hydrolysis of chiral substrates by PTE_wt (A) and PTE_G60A (B). The enantioselectivity (S_P/R_P) was calculated from the respective k_{cat}/K_M values listed in Table S3. *The catalytic efficiency for the respective R_P enantiomer could not be precisely determined and hence, a lower limit for the enantioselectivity was estimated. Detailed enzyme and substrate concentrations are listed in Table S6.

Extended text associated with Figure S3:

The hydrolysis of paraoxon (substrate I) catalyzed by PTE_wt turned out to be almost limited by the diffusion rate as evident from a k_{cat}/K_M of 8.3 x 10^7 M^{-1} s^{-1}, which is in agreement with a previously reported value of 6.4 x 10^7 M^{-1} s^{-1}.[51] Catalytic efficiencies were similarly high for the hydrolysis of the S_P enantiomer for substrates II-V (**Table S3**). The k_{cat}/K_M values of the respective R_P enantiomers were slightly lower for substrates II and III and remarkably lower for substrates IV and V, resulting in enantioselectivities of 21 (II), 15 (III), 20000 (IV), and 29000 (V) (**Table S4**, **Figure S3A**). PTE_G60A showed a catalytic efficiency for the hydrolysis of paraoxon (I) that resembles PTE_wt (**Table S3**). Catalytic efficiencies for the hydrolysis of the S_P enantiomers of substrates II and III were comparable with PTE_wt and remarkably higher than for the respective R_P enantiomers, resulting in enantioselectivities of 6200 (II) and 8100 (III). The catalytic efficiencies for the hydrolysis of the R_P enantiomers for substrates IV and V were too low to be determined, implying even higher enantioselectivities (**Table S4**, **Figure S3B**).

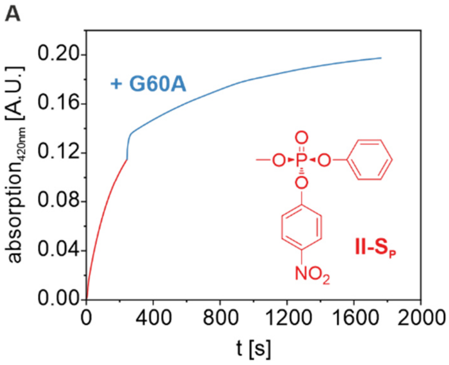

Figure S4. Complementation assay for identification of the preferentially hydrolyzed enantiomer of substrate II by PTE_I106A-H257ONBY. (A) Hydrolysis curve of 10 µM substrate II catalyzed by PTE_I106A-H257ONBY #1 (2 nM) before irradiation. The curve is mostly unaffected by addition of G60A (5 nM), implying that S_P is favored over R_P hydrolysis. **(B)**Hydrolysis curve of 10 µM substrate II catalyzed by PTE_I106A-H257ONBY #1 (0.5 nM) after irradiation at 365 nm. The noticeable jump in absorption upon addition of PTE_G60A (5 nM) indicates a preference for R_P and thus, an inversion of stereoselectivity upon irradiation.

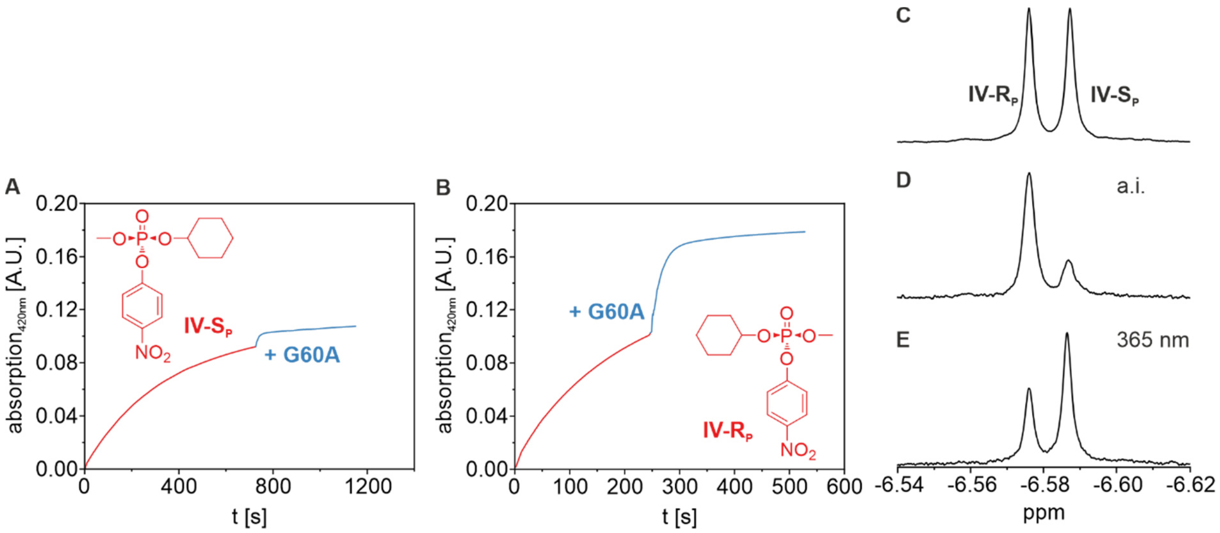

Figure S5. Complementation assay and NMR spectra for identification of the preferentially hydrolyzed enantiomer of substrate IV by PTE_I106A-F132A-H257ONBY. (A) Hydrolysis curve of 10 µM substrate IV catalyzed by PTE_I106A_F132A-H257ONBY #1 (100 nM) before irradiation. The curve is mostly unaffected by addition of G60A (15 nM), implying that S_P is favored over R_P hydrolysis. **(B)** Hydrolysis curve of 10 µM substrate IV catalyzed by PTE_I106A-F132A-H257ONBY #1 (30 nM) after irradiation at 365 nm. The noticeable jump in absorption upon addition of PTE_G60A (15 nM) indicates a preference for R_P and thus, an inversion of stereoselectivity upon irradiation. **(C)** Reference spectrum of substrate IV: Resonances for R_P and S_P were assigned with the help of the S_P-specific variant PTE_G60A. **(D)** Spectrum after incubation of substrate IV (200 µM) with 50 nM PTE_I106A-F132A-H257ONBY #2 for 3 min before irradiation. **(E)** Spectrum after incubation of substrate IV (200 µM) with 25 nM pre-irradiated PTE_I106A-F132A-H257ONBY #2 for 3.5 min. The differences in peak heights indicate distinct enantiomer preferences before and after irradiation.

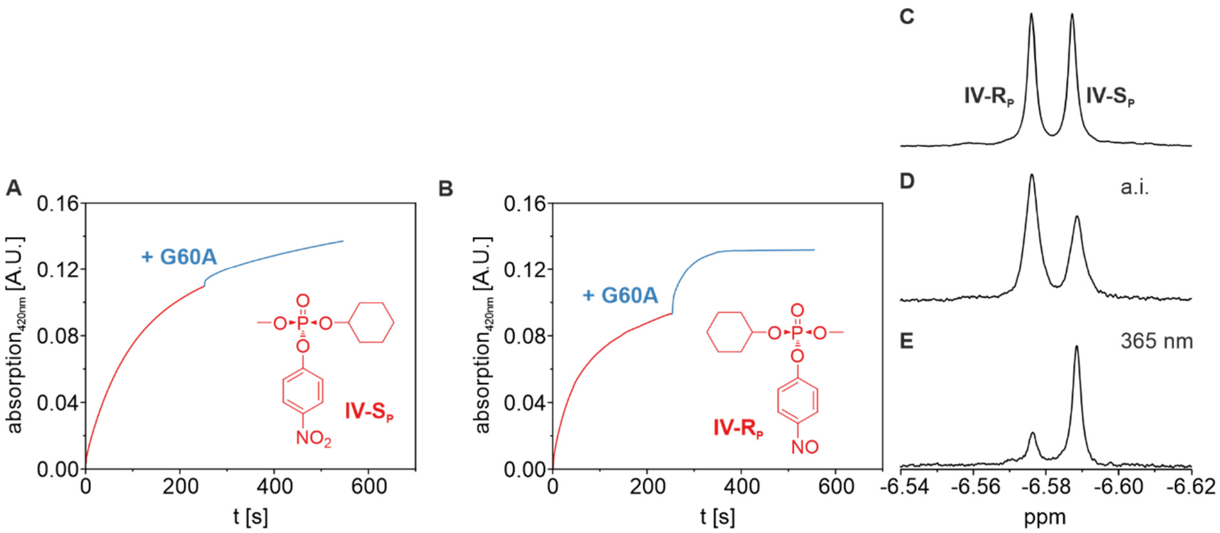

Figure S6. Complementation assay and NMR spectra for identification of the preferentially hydrolyzed enantiomer of substrate IV by PTE_I106A-F132A-S308A-H257ONBY. (A) Hydrolysis curve of 10 µM substrate IV catalyzed by PTE_I106A-F132A-S308A-H257ONBY #1 (500 nM) before irradiation. The curve is mostly unaffected by addition of G60A (5 nM), implying that S_P is favored over R_P hydrolysis. **(B)** Hydrolysis curve of 10 µM substrate IV catalyzed by PTE_I106A-F132A-S308A-H257ONBY #1 (50 nM) after irradiation at 365 nm. The noticeable jump in absorption upon addition of PTE_G60A (5 nM) indicates a preference for R_P and thus, an inversion of stereoselectivity upon irradiation. **(C)** Reference spectrum of substrate IV: Peaks for R_P and S_P were assigned with the help of the S_P-specific variant PTE_G60A. **(D)** Spectrum after incubation of substrate IV (200 µM) with 100 nM PTE_I106A-F132A-S308A-H257ONBY #2 for 5 min before irradiation (a.i.). **(E)** Spectrum after incubation of substrate IV (200 µM) with 50 nM pre-irradiated PTE_I106A-F132A-S308A-H257ONBY #2 for 2.5 min (365 nm). The differences in peak heights indicate distinct enantiomer preferences before and after irradiation.

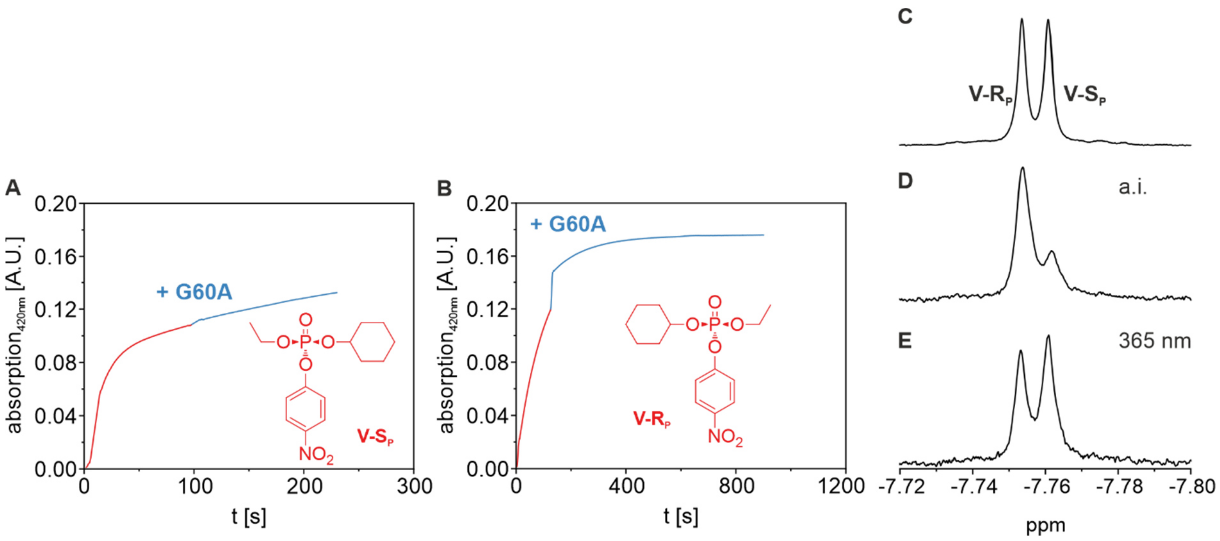

Figure S7. Complementation assay and NMR spectra for identification of the preferentially hydrolyzed enantiomer of substrate V by PTE_I106A-F132A-S308A-H257ONBY (A) Hydrolysis curve of 10 µM substrate V catalyzed by PTE_I106A-F132A-S308A-H257ONBY #1 (500 nM) before irradiation. The curve is mostly unaffected by addition of G60A (15 nM), implying that S_P is favored over R_P hydrolysis. **(B)** Hydrolysis curve of 10 µM substrate V catalyzed by PTE_I106A-F132A-S308A-H257ONBY #1 (25 nM) after irradiation at 365 nm. The noticeable jump in absorption upon addition of PTE_G60A (10 nM) indicates a preference for R_P and thus, an inversion of stereoselectivity upon irradiation. **(C)** Reference spectrum of substrate V. Peaks for R_P and S_P were assigned with the help of the S_P-specific variant PTE_G60A. **(D)** Spectrum after incubation of substrate V (200 µM) with 100 nM PTE_I106A-F132A-S308A-H257ONBY #2 for 2.5 min before irradiation (a.i.). **(E)** Spectrum after incubation of substrate V (200 µM) with 30 nM pre-irradiated PTE_I106A-F132A-S308A-H257ONBY #2 for 3.5 min (365 nm). The differences in peak heights indicate distinct enantiomer preferences before and after irradiation.

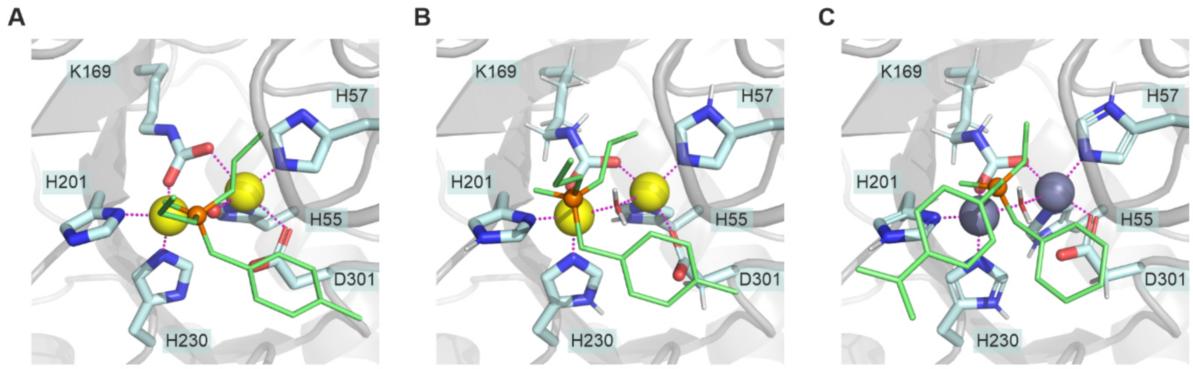

Figure S8. Ion coordination of the active site. (A) Zink ion (yellow) coordinating residues (carbon atoms cyan; oxygen atoms red; nitrogen atoms blue) and water molecule (small red sphere) as observed in the PTE X-ray structure (PDB: 1dpm)[1] with bound inhibitor (carbon atoms light green; phosphor atom orange sphere). Ion interactions are highlighted by magenta dotted lines. **(B)** Energy optimized active site of the X-ray structure with added hydrogens (light gray). **(C)** Energy optimized predicted active site architecture of PTE_I106A-H257Y with cobalt ions (dark gray) and bound substrate II (carbon atoms light green; phosphor atom orange sphere). The ion coordination sphere is the same within all structures consisting of four histidine residues, one aspartic acid, an N-formylated lysine, and one water molecule. The position of the phosphate atom of the different substrates varies by approximately 0.6 Å but is always positioned in close distance to the water molecule that is coordinated by the two metal ions. The similar ion coordination sphere of the optimized structure (B) in comparison to the X-ray structure (A) validates the derived parameters and constitutes a prerequisite for further analysis of the PTE variants.

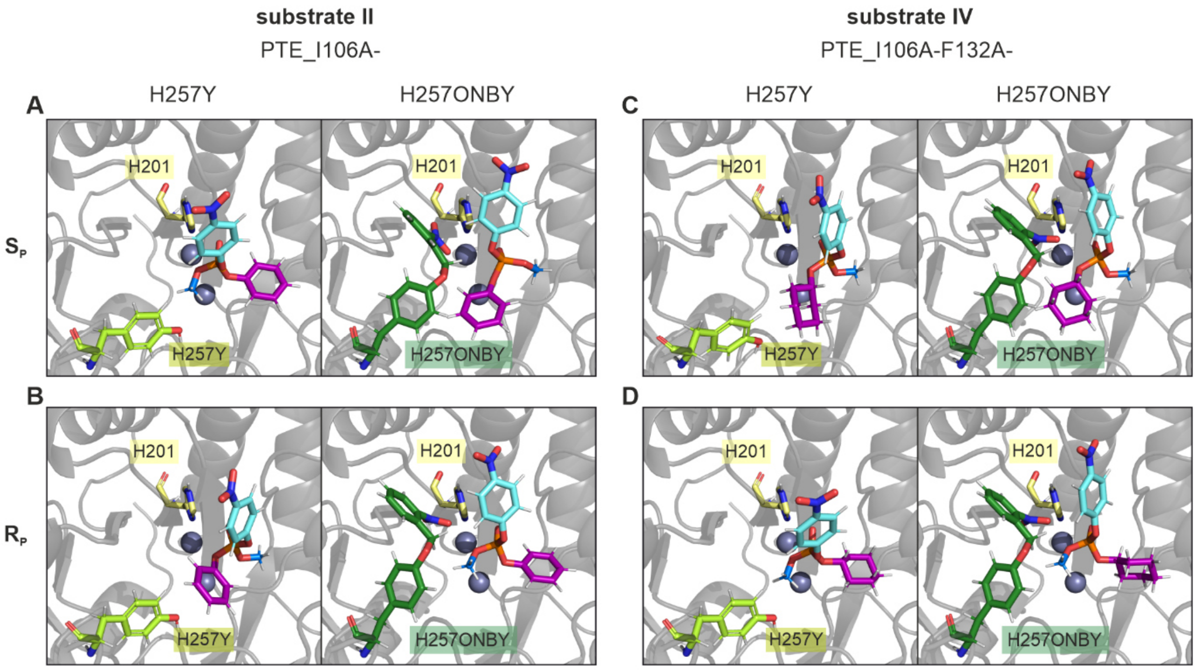

Figure S9. Predicted active site architectures of PTE_I106A-H257ONBY and PTE_I106A-F132A-H257ONBY. Comparison of predicted active site structures of PTE_I106A-H257Y and PTE_I106A-H257ONBY with bound S$_P$ **(A)** and R$_P$ enantiomer **(B)** of substrate II as well of PTE_I106A-F132A-H257Y and PTE_I106A-F132A-H257ONBY with bound S$_P$ **(C)** and R$_P$ enantiomer **(D)** of substrate IV. The cobalt ion (grey), the ion coordinating H201 (yellow), Y257 (light green), ONBY (dark green), and the substrate (colored according to Figure 2) are highlighted. In all cases the ONBY is orientated towards the H201 into the exit/entrance tunnel and the the *o*-nitrobenzyl caging group of ONBY could interact with the substrate leaving group. Both Y257 and ONBY257 could interact in the same manner with the large substituent of the substrate. All four predicted active site architectures allow binding of both enantiomers without significant differences within the accuracy of the prediction methodology.

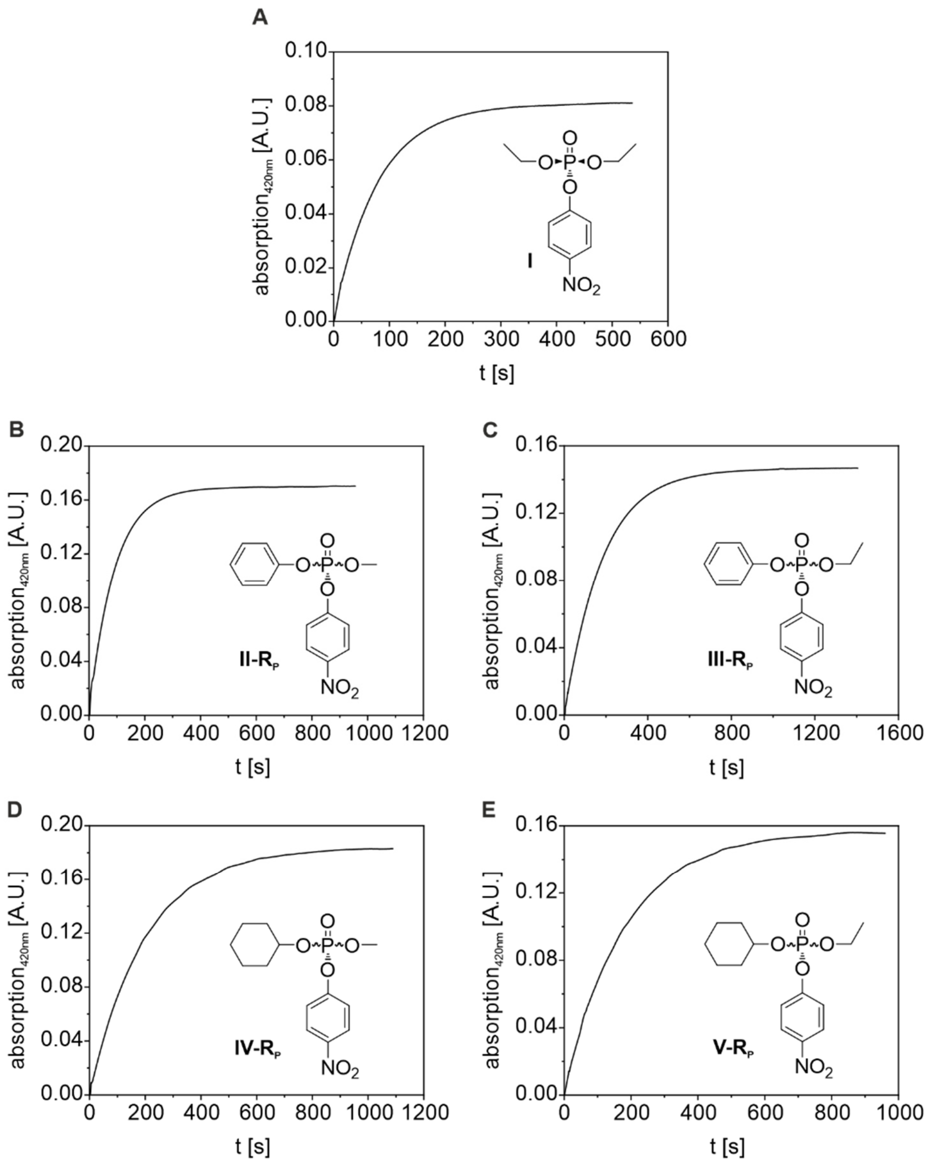

Figure S10. Chemical hydrolysis of substrates I–V. (A) Hydrolysis of 5 µM substrate I by addition of 1.5 M KOH. **(B)** Hydrolysis of 10 µM racemic substrate II by addition of 0.1 M KOH. **(C)** Hydrolysis of 10 µM racemic substrate III by addition of 0.1 M KOH. **(D)** Hydrolysis of 10 µM racemic substrate by addition of 1 M KOH. **(E)** Hydrolysis of 10 µM racemic substrate V by addition of 2 M KOH.

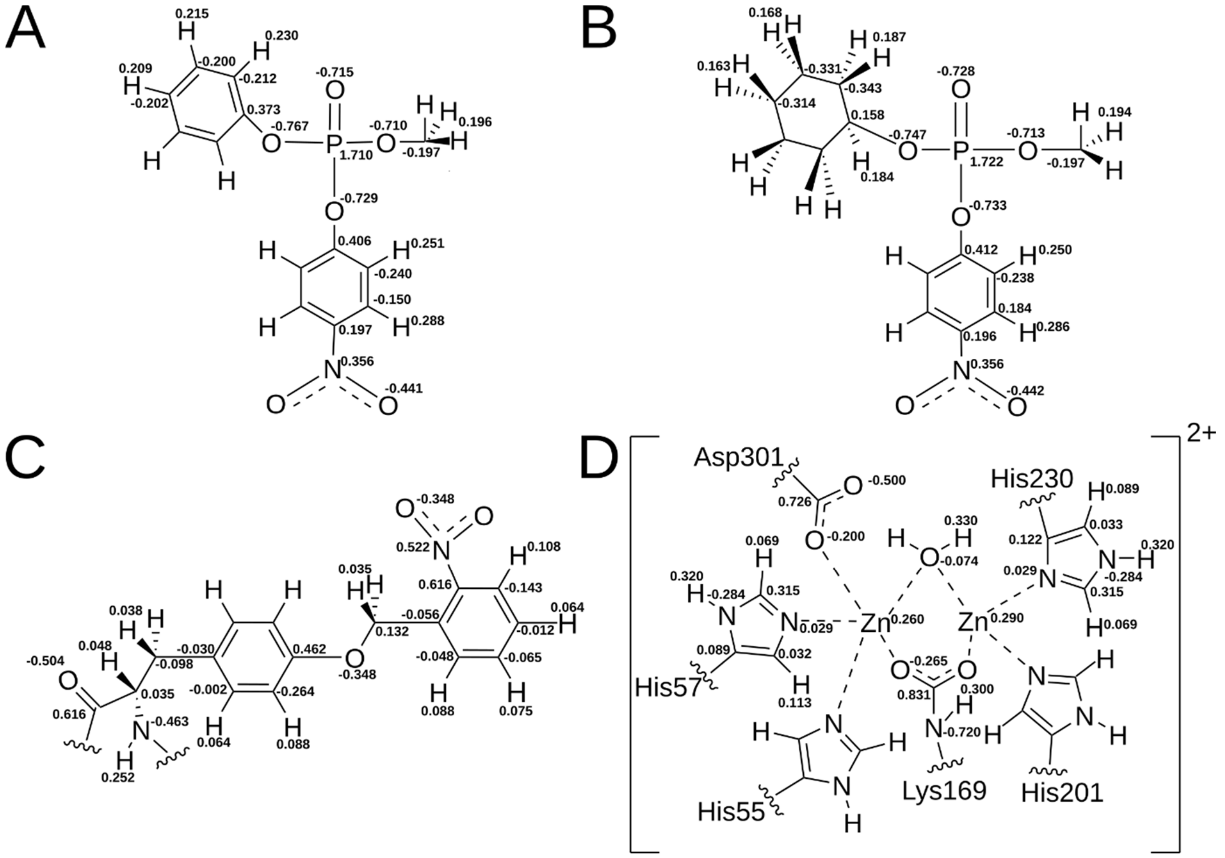

Figure S11. Charge distribution used for structure optimization. The charge distribution as used in the structure optimization is depicted for substrate II **(A)**, substrate IV **(B)**, ONBY **(C)** and for the ion complexion cluster **(D)**. For clarity, charges on chemically symmetric centers are shown only once. All omitted charges are consistent with the AMBER All-Atom force field.

Table S1. PTE variants expressed and purified in this work.

Variant	Purification steps	Final concentration [µM]	Purification yield [mg/l]
**PTE_wt**	IMAC, SEC	95	89
**PTE_G60A**	IMAC, SEC	31	78
**PTE_H257ONBY**	IMAC	17	1.2
**PTE_H257Y**	IMAC, SEC	66	18
**PTE_I106A-H257ONBY**	IMAC	37	6.3
**PTE_I106A-H257Y**	IMAC, SEC	120	64
**PTE_I106A-F132A-H257ONBY**	IMAC	54	6.8
**PTE_I106A-F132A-H257Y**	IMAC, SEC	149	177
**PTE_I106A-S308A-H257ONBY**	IMAC	58	18
**PTE_I106A-S308A-H257Y**	IMAC, SEC	105	85
**PTE_I106A-F132A-S308A-H257ONBY**	IMAC	50	15
**PTE_I106A-F132A-S308A-H257Y**	IMAC, SEC	155	169

IMAC: immobilized metal-chelate affinity chromatography; SEC: size-exclusion chromatography. Protein concentrations were determined with the Bradford assay. The yield is given as mg of pure protein per l of expression culture.

Table S2. Targeted mass spectrometric analysis (SRM) of the amino acid composition at position 257 in PTE variants.

	a.i.[a]			365 nm[b]		
**Position 257**	**ONBY [%]**	**OABY [%]**	**Tyr [%]**	**ONBY [%]**	**OABY [%]**	**Tyr [%]**
**PTE_H257ONBY**	91	0	9	0	0	100
**PTE_I106A-H257ONBY**	90	0	10	2	0	98
**PTE_I106A-F132A-H257ONBY**	96	0	4	0	0	100
**PTE_I106A-S308A-H257ONBY**	96	0	4	2	0	98
**PTE_I106A-F132A-S308A-H257ONBY**	96	0	4	0	0	100

[a]a.i. describes the "as isolated" sample, before irradiation. [b]Decaging of ONBY was performed by illumination of the sample with 365 nm for 5 min prior to sample preparation for quantitative mass spectrometry. Representative SRM traces and MS/MS spectra are shown in Figure S2.

Extended text associated with Table S2:

Before irradiation, PTE_H257ONBY and PTE_I106A-H257ONBY exhibited >89% ONBY at position 257, indicating that only a minor amount (<11%) of tyrosine is present. This may either result from co-translational misincorporation of tyrosine or due to a small extent of decaging via unintentional light exposure during sample preparation. After irradiation, PTE_H257ONBY revealed complete decaging of ONBY resulting in 100% tyrosine, whereas in PTE_I106A-H257ONBY a minor fraction of ONBY (2%) was still observed. The variants PTE_I106A-F132A-H257ONBY, PTE-I106A-S308A-H257ONBY, and PTE_I106A-F132A-S308A-H257ONBY displayed an even higher degree of ONBY incorporation (>95%) before irradiation. While after irradiation, PTE_I106A-F132A-H257ONBY and PTE_I106A-F132A-S308A-H257ONBY exhibited quantitative decaging, small traces of ONBY (2%) were still present in PTE-I106A-S308A-H257ONBY. Remarkably, no OABY was detected in any of the PTE variants, indicating that ONBY was not reduced during expression in *E. coli*. This is in accordance with other studies that have reported the absence of OABY in their samples.[73] Since ONBY in PTE is integrated within the active site it might not be as vulnerable to reductases as when incorporated into more exposed regions or at the protein surface. The considerable extent of decaging and the absence of OABY, which generally represents the amount of the protein that is not decageable, are prerequisites for effective regeneration of tyrosine at position 257 and thus, for unleashing the full potential to alter enantioselectivity.

Table S3. Catalytic efficiencies k_{cat}/K_M ($M^{-1}s^{-1}$) of PTE variants for substrates I–V.

Substrate		I	II		III		IV		V	
	state	S_P+R_P	S_P	R_P	S_P	R_P	S_P	R_P	S_P	R_P
**PTE_wt**	a.i.	8.3e7	6.9e7	3.3e6	7.2e7	4.8e6	8.6e6	4.4e2	1.2e7	4.2e2
	365 nm	2.3e7	4.7e7	3.8e6	1.2e8	6.2e6	5.2e6	4.8e2	1.2e7	5.6e2
**PTE_G60A**	a.i.	6.2e6	3.7e7	6.0e3	3.0e7	3.7e3	7.4e6	n.d.	1.7e7	n.d.
**PTE_H257ONBY**	a.i.	4.4e6	1.9e6	2.8e5	2.8e7	2.2e5	9.9e5	32.8	1.2e6	21.8
	365 nm	3.6e7	1.8e6	1.8e6	8.3e6	2.5e6	1.1e6	5.0e2	3.5e6	76.3
**PTE_H257Y**	a.i.	4.2e7	8.0e6	4.5e6	3.0e7	3.5e6	9.4e5	4.2e3	9.4e6	2.9e2
**PTE_I106A-H257ONBY #1**	a.i.	6.7e5	3.0e6	7.5e5	2.3e6	2.3e6	1.5e5	4.8e4	1.1e6	9.1e2
	365 nm	1.3e7	7.0e6	1.2e8	2.9e6	1.7e7	2.8e5	3.1e4	1.2e6	2.0e4
**PTE_I106A-H257ONBY #2**	a.i.		5.3e6	1.7e6						
	365 nm		8.1e6	4.9e7						
**PTE_I106A-H257Y**	a.i.	8.1e6	1.1e6	5.5e7	5.8e6	3.1e7	1.3e5	4.8e4	1.3e6	2.2e4
**PTE_I106A-F132A-H257ONBY #1**	a.i.	2.0e5	2.1e5	3.9e5	4.2e5	7.6e5	2.7e4	1.6e3	1.3e5	1.5e3
	365 nm	1.2e7	2.9e5	5.8e7	1.2e6	3.0e7	5.6e4	7.1e5	2.3e6	1.1e5
**PTE_I106A-F132A-H257ONBY #2**	a.i.						1.3e4	7.9e2		
	365 nm						3.4e4	3.1e5		
**PTE_I106A-F132A-H257Y**	a.i.	1.0e7	1.5e5	4.1e7	6.4e5	3.3e7	1.9e5	1.1e4	1.6e5	1.0e5
**PTE_I106A-S308A-H257ONBY**	a.i.	2.8e5	6.5e5	6.5e5	9.6e5	9.6e5	9.9e4	6.3e2	2.8e5	1.2e3
	365 nm	1.8e6	1.1e6	2.3e7	2.6e6	2.1e7	3.0e5	6.8e4	6.6e5	4.3e4
**PTE_I106A-S308A-H257Y**	a.i.	6.2e6	4.3e5	2.5e7	9.9e5	1.8e7	6.7e4	3.9e4	1.4e5	2.0e4
**PTE_I106A-F132A-S308A-H257ONBY #1**	a.i.	1.7e5	3.6e5	7.0e5	1.2e5	8.9e5	2.7e4	3.1e3	1.7e5	7.2e3
	365 nm	6.1e6	4.2e5	5.0e7	1.2e6	3.4e7	4.6e4	4.0e5	2.1e5	3.4e5
**PTE_I106A-F132A-S308A-H257ONBY #2**	a.i.						1.7e4	7.4e3	9.3e4	5.0e3
	365 nm						3.8e4	4.9e5	1.5e5	6.0e5
**PTE_I106A-F132A-S308A-H257Y**	a.i.	3.1e6	1.3e5	3.1e7	7.0e5	6.6e7	8.7e3	6.5e5	7.9e4	3.6e5

For PTE variants containing ONBY the k_{cat}/K_M values for S_P and R_P are listed before irradiation (as isolated = a.i.) and after decaging (irradiation with 365 nm). For variant-substrate combinations that showed an inversion of stereoselectivity after irradiation, biological duplicates (#1, #2) were assayed. For each of those biological duplicates, the mean of technical triplicates is shown. The reactions were carried in 100 mM Tris/HCl, pH 9.0, 0.1 mM $CoCl_2$, 25 °C. n.d.: turnover was too low to be detected.

Table S4. Stereoselectivities for the hydrolysis of chiral substrates.

Substrate<br>Variant	state	II<br>S_P/R_P	III<br>S_P/R_P	IV<br>S_P/R_P	V<br>S_P/R_P
**PTE_wt**	a.i.	21	15	2.0e4	2.9e4
	365 nm	12	19	1.1e4	2.1e4
**PTE_G60A**	a.i.	6.2e3	8.1e3	n.d.	n.d.
**PTE_H257ONBY**	a.i.	6.8	1.3e2	3.0e4	5.5e4
	365 nm	1.0	3.3	2.2e3	4.6e4
**PTE_H257Y**	a.i.	1.8	8.6	2.2e2	3.2e4
**PTE_I106A-H257ONBY #1**	a.i.	4.0	1.0	3.1	1.2e3
	365 nm	5.8e-2	0.17	9.0	60
**PTE_I106A-H257ONBY #2**	a.i.	3.1			
	365 nm	0.17			
**PTE_I106A-H257Y**	a.i.	2.0e-2	0.19	2.7	59
**PTE_I106A-F132A-H257ONBY #1**	a.i.	0.54	0.55	17	87
	365 nm	5.0e-3	4.0e-2	7.9e-2	21
**PTE_I106A-F132A-H257ONBY #2**	a.i.			16	
	365 nm			0.11	
**PTE_I106A-F132A-H257Y**	a.i.	3.7e-3	1.9e-2	17	1.6
**PTE_I106A-S308A-H257ONBY**	a.i.	1.0	1.0	1.6e2	2.3e2
	365 nm	4.8e-2	0.12	4.4	15
**PTE_I106A-S308A-H257Y**	a.i.	1.7e-2	5.5e-2	1.7	7.0
**PTE_I106A-F132A-S308A-H257ONBY #1**	a.i.	0.51	0.13	8.7	23
	365 nm	8.4e-3	3.5e-2	0.12	0.62
**PTE_I106A-F132A-S308A-H257ONBY #2**	a.i.			2.3	19
	365 nm			7.8e-2	0.25
**PTE_I106A-F132A-S308A-H257Y**	a.i.	4.2e-3	1.1e-2	1.3e-2	0.22

The enantioselectivity (S_P/R_P) before (a.i.) and after decaging (irradiation with 365 nm) was calculated from the respective k_{cat}/K_M values that are listed in Table S1. For variant-substrate combinations that showed an inversion for stereoselectivity after irradiation, the mean values of technical triplicates from biological duplicates (#1, #2) are shown. n.d.: the stereoselectivity was not obtained because the turnover of the respective R_P enantiomer was too low to be detected.

Table S5. Primers used in this work.

Mutation	Primer 1	Primer 2
**H257TAG**	GTAGAGCGCAATTGGCCTGGAAG	GGAATATGATCCAGACCAATCAGATAACCAC
**H257Y**	GTATAGCGCAATTGGCCTGGAAG	GGAATATGATCCAGACCAATCAGATAACCAC
**G60A**	CATGAACATATTTGTGCGAGCAGCGCAG	CGTCAGGGTAAAACCTGCTTCGCTAATG
**I106A**	CACCTTTGATGCGGGTCGTGATGTGAG	CTAACATCAACAATGGTACGAACACCGGC
**F132A**	TGTGGGCAGATCCGCCTCTGA	GACCGGTTGCTGCAACAATATGAACATC
**S308A**	GTTTTAGCGCCTATGTGACCAATATCATGG	CAAACAGCCAATCATTGCTCACCAGAATC
**pEVOL site 1**	AAAAAAGAAGACACGTAACTGCAGTTTCAAACG	TTTTTTGAAGACACCCATATGGGATTCCTCAAAG
**pEVOL site 2**	AAAAAAGAAGACACGTAAGTCGACCATC	TTTTTTGAAGACACCCATAGATCTAATTCCTCC

Table S6. Enzyme and substrate concentrations applied in the PTE assays.

	State	Substrate	determination of k_{cat}/K_M for	c(enzyme) [nM]	c(substrate) [µM]
**PTE_wt**	a.i.	I		0.1	10
	a.i.	II	S_P	0.5	20
	a.i.	II	R_P	5	10
	a.i.	III	S_P	0.5	20
	a.i.	III	R_P	5	20
	a.i.	IV	S_P	1	20
	a.i.	IV	R_P	2000	20
	a.i.	V	S_P	1	20
	a.i.	V	R_P	4000	10
	365 nm	I		0.5	5
	365 nm	II	S_P	0.5	10
	365 nm	II	R_P	10	10
	365 nm	III	S_P	1	10
	365 nm	III	R_P	10	10
	365 nm	IV	S_P	3	10
	365 nm	IV	R_P	6000	10
	365 nm	V	S_P	1	10
	365 nm	V	R_P	2000	10
**PTE_G60A**	a.i.	I		2	5
	a.i.	II	S_P	2	10
	a.i.	II	R_P	1000	10
	a.i.	III	S_P	1	10
	a.i.	III	R_P	1000	10
	a.i.	IV	S_P	2	10
	a.i.	IV	R_P	-	-
	a.i.	V	S_P	5	10
	a.i.	V	R_P	-	-
**PTE_H257ONBY**	a.i.	I		1	5
	a.i.	II	S_P	2	20
	a.i.	II	R_P	15	20
	a.i.	III	S_P	2	20
	a.i.	III	R_P	15	20
	a.i.	IV	S_P	10	10
	a.i.	IV	R_P	20000	10
	a.i.	V	S_P	10	10
	a.i.	V	R_P	3600	10
	365 nm	I		1	5
	365 nm	II	S_P, R_P	1	20
	365 nm	III	S_P	1	20
	365 nm	III	R_P	15	20
	365 nm	IV	S_P	10	20
	365 nm	IV	R_P	3	20
	365 nm	V	S_P	1	5
	365 nm	V	R_P	10000	10
**PTE_H257Y**	a.i.	I		0.5	5
	a.i.	II	S_P, R_P	2	5
	a.i.	III	S_P, R_P	2	10
	a.i.	IV	S_P	10	10
	a.i.	IV	R_P	5000	10
	a.i.	V	S_P	5	10
	a.i.	V	R_P	25000	10
**PTE_I106A-H257ONBY**	a.i.	I		2	5
Biol. #1 tech. #1	a.i.	II	S_P	2	10
Biol. #1 tech. #1	a.i.	II	R_P	10	10
Biol. #1 tech. #2	a.i.	II	S_P	5	10

Biol. #1 tech. #2	a.i.	II	R_P	20	5
Biol. #1 tech. #3	a.i.	II	S_P, R_P	10	10
Biol. #2 tech. #1	a.i.	II	S_P, R_P	10	10
Biol. #2 tech. #2	a.i.	II	S_P, R_P	10	10
Biol. #2 tech. #3	a.i.	II	S_P, R_P	10	10
	a.i.	III	S_P	5	10
	a.i.	III	R_P	20	5
	a.i.	IV	S_P	20	10
	a.i.	IV	R_P	1000	10
	a.i.	V	S_P	40	10
	a.i.	V	R_P	1000	10
	365 nm	I		2	5
Biol. #1 tech. #1	365 nm	II	S_P	5	10
Biol. #1 tech. #1	365 nm	II	R_P	0.5	10
Biol. #1 tech. #2	365 nm	II	S_P, R_P	2	10
Biol. #1 tech. #3	365 nm	II	S_P	5	10
Biol. #1 tech. #3	365 nm	II	R_P	0.5	10
Biol. #2 tech. #1	365 nm	II	S_P	5	10
Biol. #2 tech. #1	365 nm	II	R_P	0.5	10
Biol. #2 tech. #2	365 nm	II	S_P	5	10
Biol. #2 tech. #2	365 nm	II	R_P	0.5	10
Biol. #2 tech. #3	365 nm	II	S_P	5	10
Biol. #2 tech. #3	365 nm	II	R_P	0.5	10
	365 nm	III	S_P, R_P	2	10
	365 nm	IV	S_P	10	10
	365 nm	IV	R_P	1000	10
	365 nm	V	S_P	40	10
	365 nm	V	R_P	1500	10
**PTEI_106A-H257Y**	a.i.	I		0.5	5
	a.i.	II	S_P	5	10
	a.i.	II	R_P	0.5	10
	a.i.	III	S_P	5	10
	a.i.	III	R_P	0.5	10
	a.i.	IV	S_P	30	10
	a.i.	IV	R_P	500	10
	a.i.	V	S_P	30	10
	a.i.	V	R_P	500	10
**PTE_I106A-F132A -H257ONBY**	a.i.	I		5	5
	a.i.	II	S_P, R_P	15	10
	a.i.	III	S_P	15	10
	a.i.	III	R_P	0.5	10
Biol. #1 tech. #1	a.i.	IV	S_P	400	10
Biol. #1 tech. #1	a.i.	IV	R_P	2000	10
Biol. #1 tech. #2	a.i.	IV	S_P	400	10
Biol. #1 tech. #2	a.i.	IV	R_P	2000	10
Biol. #1 tech. #3	a.i.	IV	S_P	400	10
Biol. #1 tech. #3	a.i.	IV	R_P	2000	10
Biol. #2 tech. #1	a.i.	IV	S_P	400	10
Biol. #2 tech. #1	a.i.	IV	R_P	2000	10
Biol. #2 tech. #2	a.i.	IV	S_P	400	10
Biol. #2 tech. #2	a.i.	IV	R_P	4000	10
Biol. #2 tech. #3	a.i.	IV	S_P	400	10
Biol. #2 tech. #3	a.i.	IV	R_P	2000	10
	a.i.	V	S_P	400	10
	a.i.	V	R_P	3000	10
	365 nm	I		1	5
	365 nm	II	S_P	15	10
	365 nm	II	R_P	0.5	10
	365 nm	III	S_P	5	10
	365 nm	III	R_P	0.5	10

Biol. #1 tech. #1	365 nm	IV	S_P, R_P	100	10
Biol. #1 tech. #2	365 nm	IV	S_P, R_P	400	10
Biol. #1 tech. #3	365 nm	IV	S_P, R_P	200	10
Biol. #2 tech. #1	365 nm	IV	S_P, R_P	400	10
Biol. #2 tech. #2	365 nm	IV	S_P, R_P	400	10
Biol. #2 tech. #3	365 nm	IV	S_P, R_P	400	10
	365 nm	V	S_P	20	10
	365 nm	V	R_P	100	10
**PTE_I106A-F132A-H257Y**	a.i.	I		1	5
	a.i.	II	S_P	10	10
	a.i.	II	R_P	0.5	10
	a.i.	III	S_P	5	10
	a.i.	III	R_P	0.5	10
	a.i.	IV	S_P	100	10
	a.i.	IV	R_P	500	10
	a.i.	V	S_P, R_P	100	10
**PTE_I106A-S308A-H257ONBY**	a.i.	I		10	5
	a.i.	II	S_P, R_P	15	10
	a.i.	III	S_P, R_P	15	10
	a.i.	IV	S_P	400	10
	a.i.	IV	R_P	2000	10
	a.i.	V	S_P	200	10
	a.i.	V	R_P	1000	10
	365 nm	I		2	5
	365 nm	II	S_P	10	10
	365 nm	II	R_P	0,5	10
	365 nm	III	S_P	2	10
	365 nm	III	R_P	0.5	10
	365 nm	IV	S_P	20	10
	365 nm	IV	R_P	100	10
	365 nm	V	S_P	20	10
	365 nm	V	R_P	100	10
**PTE_I106A-S308A-H257Y**	a.i.	I		2	5
	a.i.	II	S_P	0.5	10
	a.i.	II	R_P	10	10
	a.i.	III	S_P	0.5	10
	a.i.	III	R_P	20	10
	a.i.	IV	S_P, R_P	100	10
	a.i.	V	S_P, R_P	100	10
**PTE_I106A-F132A-S308A-H257Y**	a.i.	I		0.5	5
	a.i.	II	S_P, R_P	5	10
	a.i.	III	S_P, R_P	5	10
Biol. #1 tech. #1	a.i.	IV	S_P	300	10
Biol. #1 tech. #1	a.i.	IV	R_P	500	10
Biol. #1 tech. #2	a.i.	IV	S_P	300	10
Biol. #1 tech. #2	a.i.	IV	R_P	1000	10
Biol. #1 tech. #3	a.i.	IV	S_P	300	10
Biol. #1 tech. #3	a.i.	IV	R_P	500	10
Biol. #2 tech. #1	a.i.	IV	S_P, R_P	1500	10
Biol. #2 tech. #2	a.i.	IV	S_P, R_P	1500	10
Biol. #2 tech. #3	a.i.	IV	S_P, R_P	1500	10
Biol. #1 tech. #1	a.i.	V	S_P	100	10
Biol. #1 tech. #1	a.i.	V	R_P	1000	10
Biol. #1 tech. #2	a.i.	V	S_P	100	10
Biol. #1 tech. #2	a.i.	V	R_P	500	10
Biol. #1 tech. #3	a.i.	V	S_P	100	10
Biol. #1 tech. #3	a.i.	V	R_P	500	10

Biol. #2 tech. #1	a.i.	V	S_P	150	10
Biol. #2 tech. #1	a.i.	V	R_P	1500	10
Biol. #2 tech. #2	a.i.	V	S_P	150	10
Biol. #2 tech. #2	a.i.	V	R_P	1500	10
Biol. #2 tech. #3	a.i.	V	S_P	150	10
Biol. #2 tech. #3	a.i.	V	R_P	1500	10
	365 nm	I		10	5
	365 nm	II	S_P	2	10
	365 nm	II	R_P	0.5	10
	365 nm	III	S_P	10	10
	365 nm	III	R_P	1	10
Biol. #1 tech. #1	365 nm	IV	S_P	500	10
Biol. #1 tech. #1	365 nm	IV	R_P	50	10
Biol. #1 tech. #2	365 nm	IV	S_P	500	10
Biol. #1 tech. #2	365 nm	IV	R_P	50	10
Biol. #1 tech. #3	365 nm	IV	S_P	500	10
Biol. #1 tech. #3	365 nm	IV	R_P	50	10
Biol. #2 tech. #1	365 nm	IV	S_P	50	10
Biol. #2 tech. #1	365 nm	IV	R_P	500	10
Biol. #2 tech. #2	365 nm	IV	S_P	50	10
Biol. #2 tech. #2	365 nm	IV	R_P	500	10
Biol. #2 tech. #3	365 nm	IV	S_P	50	10
Biol. #2 tech. #3	365 nm	IV	R_P	500	10
Biol. #1 tech. #1	365 nm	V	S_P, R_P	50	10
Biol. #1 tech. #2	365 nm	V	S_P, R_P	50	10
Biol. #1 tech. #3	365 nm	V	S_P, R_P	50	10
Biol. #2 tech. #1	365 nm	V	S_P, R_P	50	10
Biol. #2 tech. #2	365 nm	V	S_P, R_P	50	10
Biol. #2 tech. #3	365 nm	V	S_P, R_P	50	10
**PTE_I106A-F132A-S308A-H257Y**	a.i.	I		3	5
	a.i.	II	S_P	10	10
	a.i.	II	R_P	1	10
	a.i.	III	S_P	10	10
	a.i.	III	R_P	1	10
	a.i.	IV	S_P	500	10
	a.i.	IV	R_P	50	10
	a.i.	V	S_P, R_P	50	10

Equation S1. Rearrangement of the Michaelis-Menten equation assuming that K_M >> [S].

$$v = v_{max} \cdot \frac{[S]}{[S] + K_M}$$

$$v = [E_0] \cdot k_{cat} \cdot \frac{[S]}{[S] + K_M}$$

$$-\frac{d[S]}{dt} = [E_0] \cdot k_{cat} \cdot \frac{[S]}{[S] + K_M}$$

$$-\frac{d[S]}{dt} = [E_0] \cdot k_{cat} \cdot \frac{[S]}{K_M}$$

$$\frac{d[S]}{[S]} = -\frac{k_{cat}}{K_M} \cdot [E_0] \cdot dt$$

$$\int_{[S_0]}^{[S]} \frac{d[S]}{[S]} = \int_{t=0}^{t} -\frac{k_{cat}}{K_M} \cdot [E_0] \cdot dt$$

$$\ln\left(\frac{[S]}{[S_0]}\right) = -\frac{k_{cat}}{K_M} \cdot [E_0] \cdot (t - t_0) + C$$

$$\ln\left(\frac{[S]}{[S_0]}\right) = -\frac{k_{cat}}{K_M} \cdot [E_0] \cdot t + C$$

$$\frac{[S]}{[S_0]} = e^{\left(-\frac{k_{cat}}{K_M} \cdot [E_0] \cdot t\right)} + C$$

$$[S] = [S_0] \cdot e^{\left(-\frac{k_{cat}}{K_M} \cdot [E_0] \cdot t\right)} + C$$

v : reaction velocity [M s^{-1}]
v_{max} : maximum reaction velocity [M s^{-1}]
k_{cat} : turnover number [s^{-1}]
$[E_0]$: initial enzyme concentration [M]
$[S]$: substrate concentration [M]
K_M : Michaelis constant [M]
t : time [s]

Reference

(1) Vanhooke, J. L.; Benning, M. M.; Raushel, F. M.; Holden, H. M. Three-dimensional structure of the zinc-containing phosphotriesterase with the bound substrate analog diethyl 4-methylbenzylphosphonate. *Biochemistry* **1996**, *35* (19), 6020–6025.
